# Molecular dynamics simulation‐guided toehold mediated strand displacement probe for single‐nucleotide variants detection

**DOI:** 10.1002/EXP.20210265

**Published:** 2022-01-24

**Authors:** Linghao Zhang, Jing Chen, Mengya He, Xin Su

**Affiliations:** ^1^ College of Life Science and Technology Beijing University of Chemical Technology Beijing China

**Keywords:** dynamic DNA nanotechnology, molecular dynamic simulation, oxDNA, SARS‐CoV‐2, single‐nucleotide variants, toehold mediated strand displacement

## Abstract

Single nucleotide variant (SNV) has become an emerging biomarker for various diseases such as cancers and infectious diseases. Toehold‐mediated strand displacement (TMSD), the core reaction of DNA nanotechnology, has been widely leveraged to identify SNVs. However, inappropriate choice of mismatch location results in poor discrimination ability. Here, we comprehensively investigate the effect of mismatch location on TMSD kinetics by molecular dynamic simulation tool oxDNA through umbrella sampling and forward flux sampling disclosing that mismatches at the border of the toehold and branch migration domain yield the lowest TMSD reaction rate. Nine disease‐related SNVs (*SARS‐CoV‐2‐D614G*, *EGFR‐L858R*, *EGFR‐T790M*, *KRAS‐G12R*, etc.) were tested experimentally showing a good agreement with simulation. The best choice of mismatch location enables high discrimination factor with a median of 124 for SNV and wild type. Coupling with a probe‐sink system, a low variant allele frequency of 0.1% was detected with 3 S/N. We successfully used the probes to detect SNVs with high confidence in the PCR clones of constructed plasmids. This work provides mechanistic insights into TMSD process at the single‐nucleotide level and can be a guidance for the design of TMSD system with fine‐tuning kinetics for various applications in biosensors and nanotechnology.

## Graphical abstract

This work provides mechanistic insights into toehold‐mediated strand displacement process at the single‐nucleotide level through molecular dynamics simulation. Mismatches at the border of the toehold and branch migration domain yield the lowest reaction rate. Based on the simulation results, single nucleotide variants of disease‐related genes were detected with high specificity.

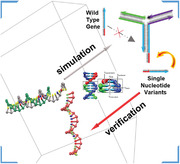

## 1 INTRODUCTION

Single nucleotide variation (SNV) arises frequently in human genome, of which the average density is calculated to be 1/1910 bases on average.^[^ ^1^ ^]^ SNVs are promising biomarkers both clinically and biologically, as single base differences in nucleic acid sequences can lead to profound biological and clinical consequences.^[^ ^2^ ^]^ SVNs form the genetic basis for a variety of human diseases or can confer drug resistance to pathogenic bacteria or viruses.^[^ ^3^ ^]^ Mutated genes in cancer, for example, are likely to increase drug resistance.^[^ ^4^ ^]^ The *SARS‐CoV‐2* with *D614G* mutation is more replicative and more easily transmitted.^[^ ^5^ ^]^ Accurate identification of SNV is of great significance for advanced diagnostics and fundamental biological research. Although the fully complementary hybridization is energetically favorable rather than mismatch‐contained hybridization, the thermodynamic gain of many correctly paired bases can easily overcome the thermodynamic penalty of a single mismatch.^[^ ^6^ ^]^ This results in low discrimination capability for SNV. Accordingly, the development of sensitive, specific, rapid, and economical methods for SNVs analysis is urgently needed.

Allele‐specific hybridization has been integrated with various signaling approaches, including fluorescence,^[^ ^7^ ^]^ solid‐state nanopores,^[^ ^8^ ^]^ single‐molecule platform,^[^ ^7^, ^9^ ^]^ microfluidic platform,^[^ ^10^ ^]^ electrochemistry,^[^ ^11^ ^]^ biosensor^[^ ^12^ ^]^ for SNV detection. Although these methods enable rapid, portable, sensitive detection for nucleic acids, the specificity is not high to fulfill the requirement of detecting a low variant allele frequency (VAF). Toehold‐mediated strand displacement (TMSD), a dynamic DNA reaction at nanoscale, is sensitive to single nucleotide mismatch and the output is a single strand which can participate in a variety of downstream reactions to devote to signal amplification.^[^ ^13^ ^]^ In a typical TMSD, the invader strand binds to the toehold domain to initiate branch migration thereby releasing the incumbent strand. The presence of mismatch between invader strand and template strand would reduce the kinetics of strand displacement. This feature makes TMSD a powerful tool to develop SNV detection methods.^[^ ^14^ ^]^ However, inappropriate choice of mismatch location results in unsatisfied discrimination ability, so far, there is lack of comprehensive study on the effect of mismatch location.^[^ ^15^ ^]^ oxDNA is a simplified coarse‐grained model code package to simulate the process of dynamic DNA interaction. It has been utilized to simulate TMSD and explore its energy landscape.^[^ ^16^ ^]^ This platform holds great potentials to provide guidance for the choice of mismatch location in TMSD.

**SCHEME 1:**
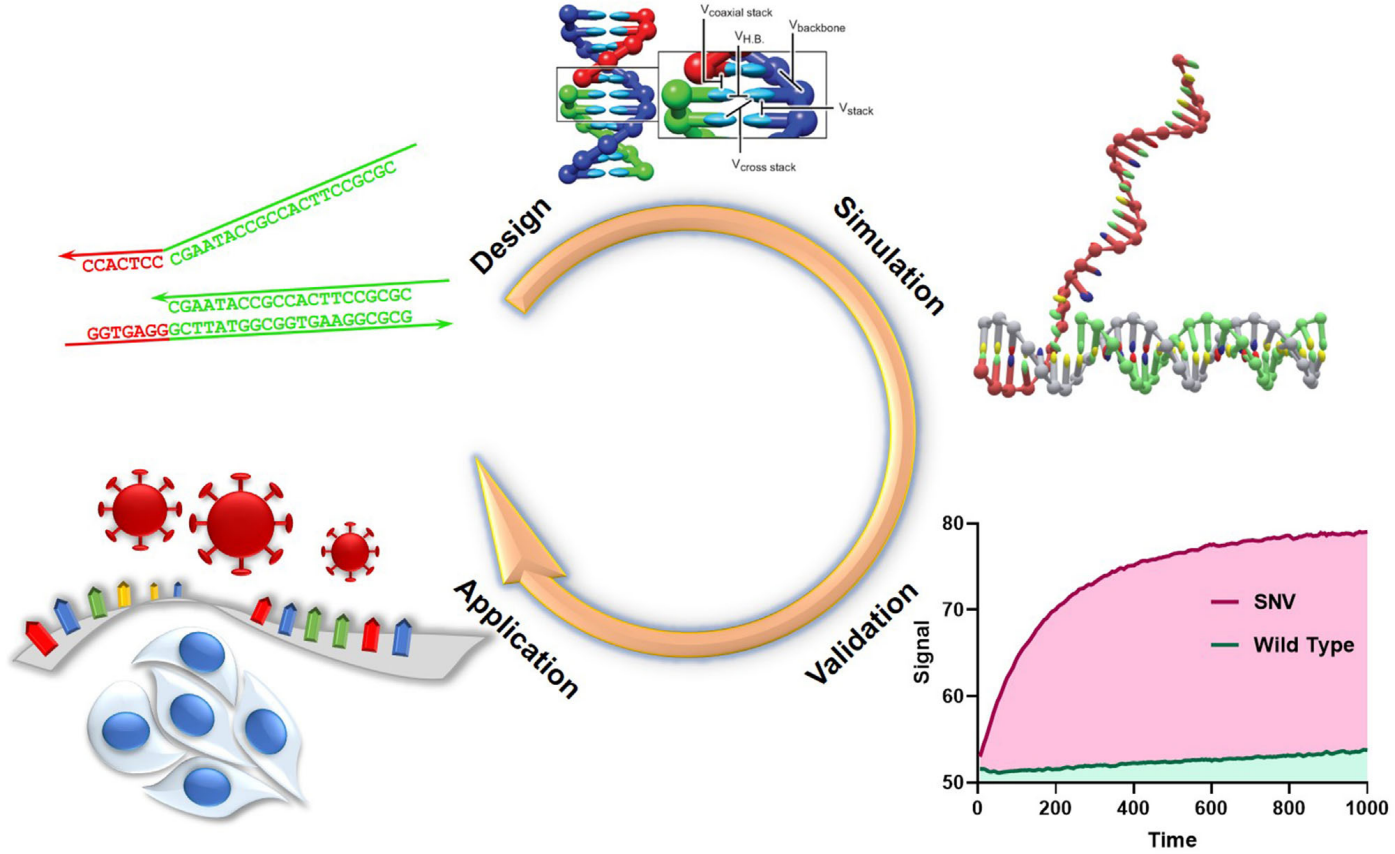
MD Simulation‐guided TMSD probe design for SNVs detection. The choice of mismatch location in TMSD guided by oxDNA simulation enables excellent discrimination of SNV and wild type (WT). The probes were finally applied to detect SNVs in cancer‐related genes and SARS‐CoV‐2

Herein, we investigated the effect of mismatch location on TMSD kinetics by oxDNA simulation via umbrella sampling (US) and forward flux sampling (FFS). We for the first time found that mismatches at the border of the toehold domain and branch migration domain exhibit more significant effect on kinetics than the other positions. This observation was experimentally verified by 9 disease‐related SNVs (Scheme 1). Border mismatches render high discrimination factor (DF) with a median of 124 which is superior than currently reported results.^[^ ^17^ ^]^ Probe‐sink system^[^ ^18^ ^]^ was employed to further enhance DF allowing for detecting VAF down to 0.1%. We successfully used the probes to detect SNVs of SARS‐CoV‐2 and cancer genes with high confidence in plasmid clones (Scheme 1). Molecular dynamic (MD) simulation provides new insights into TMSD kinetics. The results hold great potentials not only in molecular diagnostics but also in various applications of dynamic DNA nanotechnology.

## 2 RESULTS AND DISCUSSION

### 2.1 Thermodynamics of mismatch‐contained toehold‐mediated strand displacement

**FIGURE 1:**
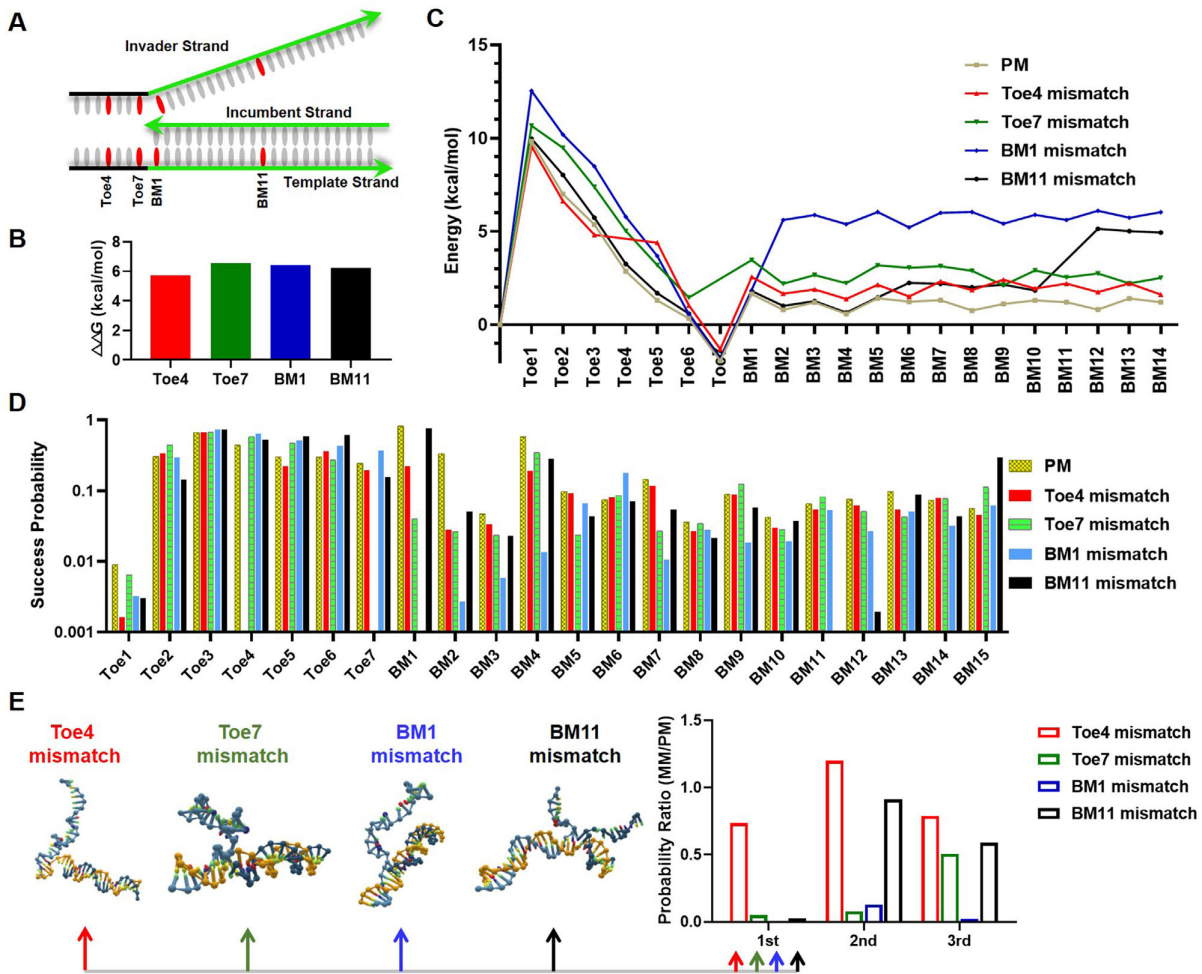
MD simulation of mismatch‐contained TMSD. (A) The choices of mismatch positions of TMSD in the model sequence. The toehold and branch migration domain are 7 and 21 nt, respectively. (B) The prediction of differential free energy (△△*G*) of MM‐TMSD versus PM‐TMSD by NUPACK (for △*G* see Table S2 in the Supporting Information). (C) Simulation of free energy landscapes for PM‐TMSD and MM‐TMSDs through US by oxDNA. (D) The success probabilities to cross interfaces in TMSD by FFS simulation. (E) The success probability ratio of MM‐TMSD and PM‐TMSD at the mismatches’ downstream neighboring bases. The conformations of the hybridization of the first downstream bases of Toe4, Toe7, BM1, and BM11 mismatches are shown. The other conformations are shown in Figure S1 in the Supporting Information

Model sequences were used to study the effect of mismatch on TMSD reactions in which the toehold domain and the branch migration domain are 7 and 21 nt, respectively. Four positions, the middle of toehold domain (Toe4), the border bases of toehold and branch migration domain (Toe7 and BM1), and the middle of branch migration domain (BM11) were chosen as representatives (Figure 1A). In fact, such TMSD configuration and mismatch positions were commonly found in TMSD probes for SNV detection.^[^ ^19^ ^]^ TMSD is a thermodynamically favorable process. Free energy of TMSD △*G* can be calculated as the established model by using a website service NUPACK.^[^ ^20^ ^]^ △△*G* was defined as the free energy change difference between perfectly matched TMSD (PM‐TMSD) and single mismatched TMSD (MM‐TMSD) (for details see Table S2 in the Supporting Information). As shown in Figure 1B, there is no significant difference in △△*G* for the G‐G mismatches at different positions. In other words, mismatches at different positions yield almost identical free energy change in TMSD. Therefore, dissecting TMSD at the single‐nucleotide level can be helpful to study the effect of mismatch positions.

We explored the energy landscape of PM‐TMSD and MM‐TMSD by oxDNA platform. oxDNA is a simulation code originally developed to implement the coarse‐grained DNA model, which has grown in popularity in recent years and is widely used to prototype new nucleic acid nanostructure designs, model biophysics of DNA/RNA processes, and rationalize experimental results.^[^ ^21^ ^]^ Each nucleotide in this model is represented as a rigid body with specific interaction sites that approximate the geometry and interactions of atoms (>20) that make up each nucleotide in oxDNA.^[^ ^22^ ^]^ oxDNA can be used to simulate the steps involved in DNA and RNA strand displacement at a single‐base level and offer a good representation of strand displacement.^[^ ^16^, ^23^ ^]^ A built‐in algorithm in oxDNA, US, can extract the duration of various states in dynamic simulation according to setting order parameters. We obtained this information of the intermediate states in TMSD given by order parameters file and weight file of US (Tables S3–S7 in the Supporting Information), then the relative free energy between each intermediate state were obtained by the equation △△*G* = ‐*RT*ln*K*_eq_. The equilibrium constant *K*_eq_ in the equation was replaced by the ratio of dwell time about two adjacent states.^[^ ^23c^ ^]^ The longer the state lasts, the more likely it is to occur indicating the state is in a lower energy. Thus, the energy landscape of TMSDs can be obtained (Figure 1C). The dynamic simulation started from the initial state which was confined in a box containing invade strand and target strand with a distance more than 4.0 (1.0 = 0.8518 nm). Bases are not paired until the distance is less than 1. The landscape indicates that initial energy barrier can be overcome by the base hybridization at toehold domain, energy drops to a valley while toehold binding completes. In branch migration domain, energy exhibits fluctuation pattern attributed to the dynamic of base association and dissociation (Figure 1C). MM‐TMSDs exhibit their own additional energy barriers at the corresponding mismatch positions. We found there is no significant difference between four kinds of energy barriers (2–3 kcal mol^−1^) which couldn't contribute to distinguish single nucleotide variant. Specifically, BM1 creates the additional free‐energetic penalty of 8.4 kcal mol^−1^, which is significantly higher than the other types of MM‐TMSD (Figure 1C). Accordingly, BM1 might show remarkable effect on TMSD reaction.

### 2.2 Toehold‐mediated strand displacement kinetics simulation by forward flux sampling

Owing to the different barriers created by mismatches in energy landscape, we speculated that mismatches at different positions may exhibit different effects on TMSD kinetics. The algorithm FFS in oxDNA, which describes dynamic DNA interactions, was employed to simulate the effect of mismatch position on TMSD kinetics.^[^ ^16^, ^23^, ^24^ ^]^ In FFS simulation, TMSD process is divided into individual interfaces, each interface represents a single base. The success probabilities of crossing each interface were recorded by available python scripts and order parameters file (Figure 1D, Table S8 in the Supporting Information).

The success probability of crossing the first base Toe1 is low because of the initial thermodynamic barrier. Subsequently, the success probabilities of crossing the rest interfaces in the toehold domain are significantly promoted owing to continuous energy decline. In the stage of branch migration, the variation of success probabilities can be attributed to the reverse reaction (Figure 1D). The success probabilities to cross mismatches and their downstream bases are significantly lower than that to across paired bases. The mismatches’ downstream bases are critical for the success of the entire TMSD. The datasets shown in Figure 1E were defined as the success probability ratio (PR) of MM‐TMSD and PM‐TMSD at the mismatches’ downstream neighboring bases, thereby, lower PR is referred to stronger discrimination capability for MM‐TMSD and PM‐TMSD. The mismatches at Toe7 and BM1 exhibit remarkable ability to inhibit displacement of the first downstream base of mismatches with the probability ratios of 0.05 and 0.008, respectively. Similarly, they also significantly affect their downstream second and third bases. This result is consistent with the results of the energy landscape. The probability ratios of the downstream bases of Toe4 mismatch are relatively higher than those of Toe7 and BM1 mismatches. Because the toehold bonding is a process of energy decline, the base pairing in the toehold domain was less affected by the presence of mismatch. The mismatch at BM11 shows the slightest effect toward its downstream bases, the second and third bases even show close probability as PM‐TMSD (Figure 1E). In the middle of branch migration like BM11, reverse reaction is more prominent. The mismatch not only prevents the forward reaction but also the reverse reaction, which accounts for the high probabilities of the second and third bases. Single mismatch is no longer an effective limiting factor in the middle and distal segments of branch migration. By the comprehensive analysis, it is concluded that Toe7 and BM1, the border bases of toehold and branch migration domain, are the most efficient mismatch positions to inhibit TMSD.

### 2.3 Experimental validation of the simulation results

**FIGURE 2:**
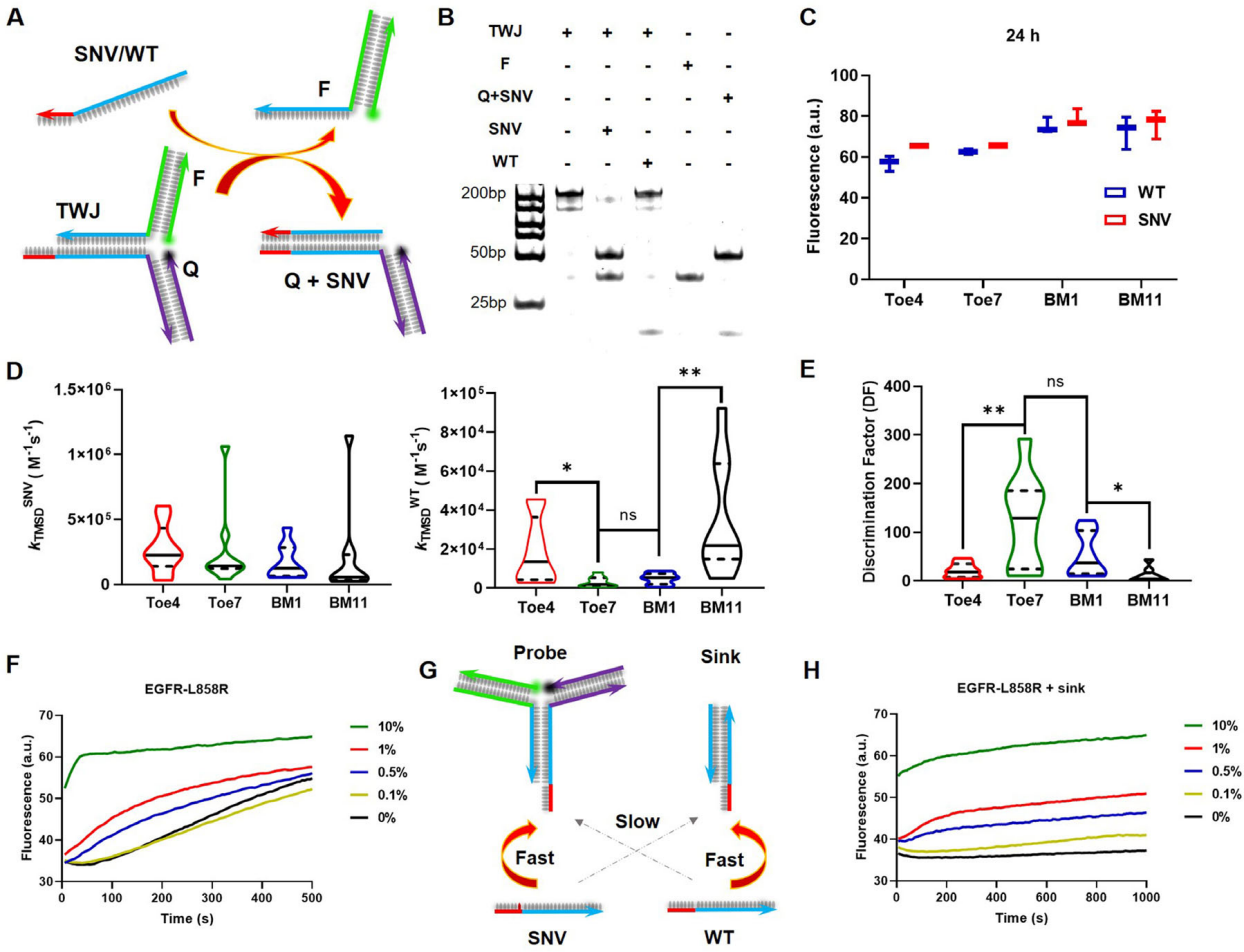
Experimental verification of the results by simulation and VAF detection. (A) TWJ probe for SNV detection. The probes are fully complementary with SNV but forms single mismatch with WT. Each gene is adopted with four probes bearing mismatch at Toe4, Toe7, BM1, and BM11, respectively. (B) Verification of TMSD products by PAGE. Mismatch was located at Toe7. (C) Fluorescence intensities of the four TMSD probes after 24 h. (D) Experimental TMSD rate constants kTMSD of SNV and WT. (E) Discrimination factors for all tested genes. (*p < 0.05, **p < 0.01). In all violin charts of panel d and e, the horizontal solid line and dashed line represent the medians and quartiles. (F) Fluorescence curves of TMSD probes (Toe7 mismatch) in the presence of different percentages of SNV strands. (G) Scheme of probe‐sink system. The probe a TWJ structure is fully complementary with SNV and forms single mismatch with WT. The sink a linear TMSD structure is fully complementary with WT and forms single mismatch with SNV. (H) Fluorescence curves of probe‐sink systems (Toe7 mismatch) in the presence of different percentages of SNV strands

Next, we used nine disease‐related SNVs (*SARS‐CoV‐2‐D614G*, *SARS‐CoV‐2‐N501Y*, *EGFR‐L858R*, *EGFR‐T790M*, *NRAS‐G12C*, *KRAS‐G12R*, *PIK3CA‐H1047R*, *STK11‐F354L*, *TP53‐Y220C*) to validate the simulation results. Three‐way junction (TWJ) structure was used. In TWJ, universal fluorophore‐labeled and quencher‐labeled strands can be shared by different genes. The target sequences act as invader strand binds to the toehold domain and release fluorescent incumbent strands by branch migration, resulting in fluorescence dequenching (Figure 2A). To view the mutation via “turn on” approach, TMSD probes were designed fully complementary with SNV strand, but formed single mismatch with WT strand. Each gene was adopted with four TMSD probes. Different TMSD probes formed mismatches with WT strand at the above simulated positions, Toe4, Toe7, BM1, and BM11.

TMSD reactions were characterized by polyacrylamide gel electrophoresis (PAGE). Reaction products of 10 min are shown in Figure 2B. When mismatch is located at the Toe7 position, SNV exhibits significantly higher TMSD yield than WT. However, from the fluorescence intensity, SNV and WT yield almost identical TMSD products after 24 h regardless of the mismatch position (Figure 2C). These results prove that the presence of mismatch mainly affects TMSD kinetics rather than thermodynamics. The isothermal fluorescence kinetics is shown in Figures S2–S10 in the Supporting Information, and all datasets are fitted with the second order kinetic to generate experimental reaction constants (Figure S11 in the Supporting Information). We defined *k*_TMSD_^SNV^ and *k*_TMSD_^WT^ as the reaction constants for SNV and WT, respectively, and defined discrimination factor (DF) as the ratio of *k*_TMSD_^SNV^ and *k*_TMSD_^WT^. The distribution of reaction constants of the nine genes against TMSD probes are shown in Figure 2D, in which the horizontal solid line and dashed line represent the medians and quartiles. Briefly, the probes yield relatively lower *k*_TMSD_^WT^ if the mismatches located at Toe7 and BM1. The median DFs of Toe4, Toe7, BM1, and BM11 are 18.5, 124, 37.1, and 10.7, respectively (Figure 2E). Toe7 and BM1 yield significantly high median DF values making these two sites as the best choices for mismatch location. These results are perfectly consistent with the simulation results. The experimental DF values are superior or comparable with recently reported results.^[^ ^13^, ^15^, ^17^, ^25^ ^]^ Although Toe4 has been wildly used in SNV detection,^[^ ^26^ ^]^ our study discloses that Toe7 and BM1 can offer better discrimination effect than Toe4.

### 2.4 Detection of low variant allele frequency in single nucleotide variant

The VAF corresponding to the fraction of sequencing reads harboring the mutation varies in clinical samples. *EGFR* mutant is an important cancer mutant factor with a high mutant rate, the detection of *EGFR* mutant has important clinical significance.^[^ ^27^ ^]^ The VAF of *T790M* in *EGFR* gene, is found as approximately 50% in clinical TKI‐relapsed patients samples.^[^ ^28^ ^]^ Studies have shown that *D614G* mutant enhances the infectivity of *COVID‐19*.^[^ ^5^ ^]^ *D614G* mutant type was detected as 29% in *COVID‐19* patient samples.^[^ ^29^ ^]^ Therefore, it is desired that SNV probes is capable of detection low concentrations of SNVs in the presence of high concentrations of WTs. According to simulation results and experimental validation, we demonstrated such capability of the simulated TMSD probes by testing low VAF of *SARS‐CoV‐2‐D614G*, *EGFR‐L858R*, and *EGFR‐T790M* The TMSD probes which forms mismatch with WT sequences at Toe7 position the border of toehold and branch migration domain. In all assays, the total concentration of target sequences was fixed at 500 nm, VAF varies from 0.1% to 10%. As shown in Figure 2F and Figure S12A in the Supporting Information, 0.5% VAF can be detected with 3 S/N, and signal increases as a function of VAF from 0.5% to 10%.

To further enhance the detection capability of low VAF, we introduced probe‐sink system.^[^ ^25^ ^]^ As shown in Figure 2G, the probe and sink were designed TMSD probes which are perfectly complementary with SNV and WT, respectively. The presence of sink can effectively reduce the available WT thereby enhancing the reaction between SNV and probe. The prerequisite of successful probe‐sink system is that TMSD probe has a high DF toward SNV because low DF can induce cross‐talks of probe/WT and sink/SNV.^[^ ^30^ ^]^ Our MD‐simulated TMSD probes pose high DF which is beneficial for operating probe‐sink system. In our case, the probe is TWJ form, and the sink is double‐stranded form (Figure 2G). With the support of sink, VAF of the three genes can be detected down to 0.1% with 3 S/N, that meets the requirement of clinical low abundant VAF detection (Figure 2H and Figure S12B in the Supporting Information).^[^ ^31^ ^]^

### 2.5 Multiplexed detection

**FIGURE 3:**
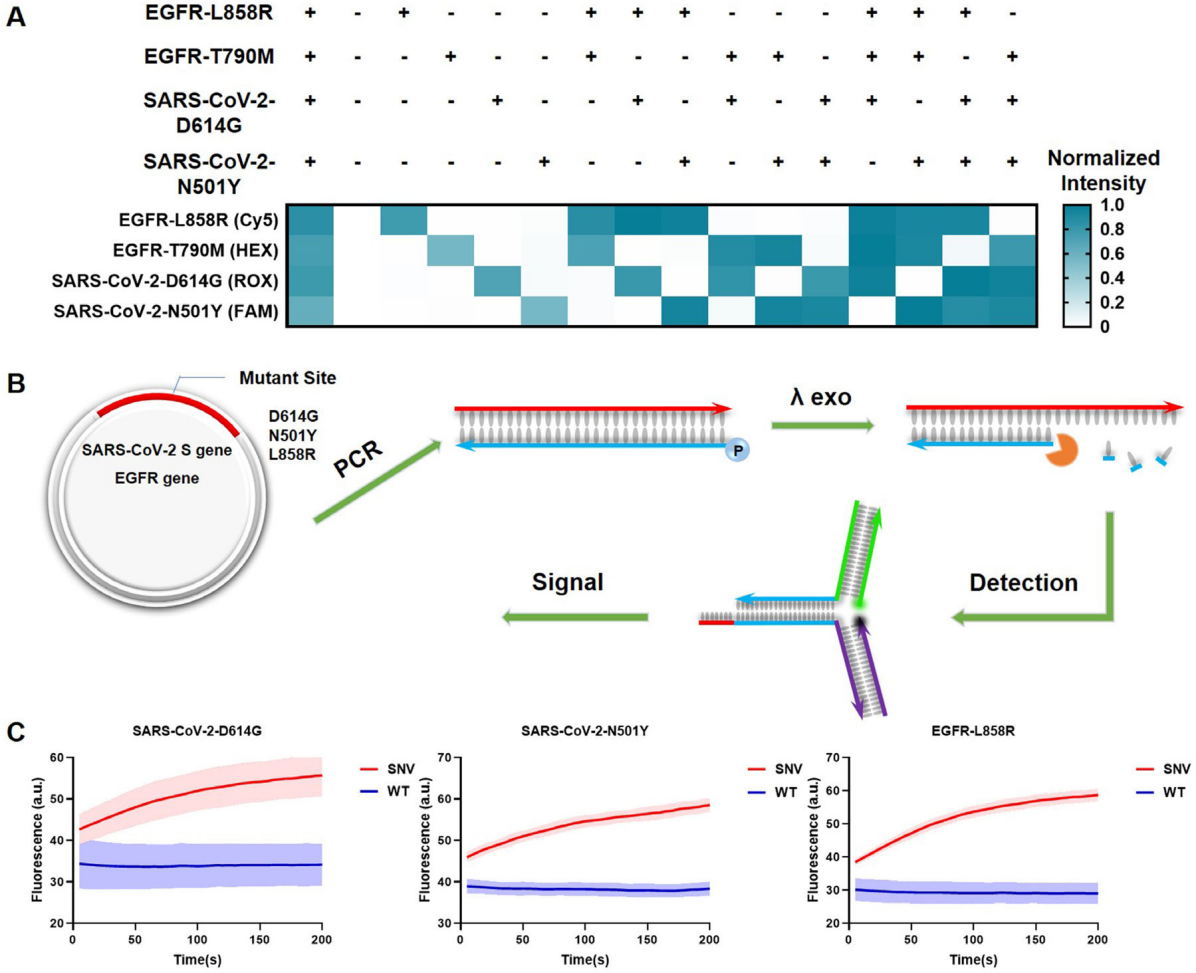
Multiplexed detection and SNV detection in plasmid clones. (A) Results of multiplexed detection. 'Plus’ and ‘minus’ symbols represent the SNV and WT of the corresponding genes. The raw fluorescence curves are shown in Figure S13 in the Supporting Information. The fluorescence intensities of the products and reactants of TMSD reaction were normalized as ‘1′ and ‘0′ for each color. (B) Procedures for SNV detection in plasmid. Regions of interest are amplified by PCR, the 5′ end of the reverse primers are phosphorylated. λ Exo was used to generated single‐stranded DNA targets. (C) Fluorescence curves of SNV detection in PCR amplicons. All experiments were performed with three replicates

Multiple SNVs are commonly found in cancer‐related genes and virus.^[^ ^32^ ^]^ Simultaneous detection of multiple mutations significantly enhances diagnostics accuracy. Therefore, the orthogonality of gene probes can avoid the possibility of false positives in mixed detection. We tested SNVs in four different genes by labeling the TMSD probes with different kinds of fluorophore (FAM, HEX, ROX, and Cy5). The presence of DNA from different combinations of SNVs could be inferred from the corresponding normalized signal intensity. To our satisfaction, little or no signal was observed in the absence of the corresponding SNVs. Therefore, highly orthogonal detection was achieved (Figure 3A).

### 2.6 Single nucleotide variant detection in plasmid clones

To demonstrate the feasibility of our TMSD probe for clinical samples, we constructed plasmid with S gene in *SARS‐CoV‐2* and two cancer‐related SNV sites (*D614G* and *N501Y*) to mimic the corresponding nucleic acid targets in clinical samples. The same is for *EGFR* gene and the SNV site (*L858R*). To generate single‐stranded amplicons, the 5′ end of the reverse primer was phosphorylated and λ Exonuclease (λ Exo) can recognize and digest the phosphorylated strand thereby generating single‐stranded amplicons (Figure 3B). Gel electrophoresis confirms the generation of single‐stranded amplicon of all genes (Figure S14 in the Supporting Information). Then we detected these products by the TMSD probes (mismatch located at Toe7), as shown in Figure 3C, SNV and WT about these three genes are effectively distinguished.

## 3 CONCLUSION

In summary, we utilized MD simulation platform oxDNA to guide the design of the TMSD probe for highly specific SNV detection. The energy landscape and forward displacement probability disclose that mismatches located at the border of toehold and branch migration domain show a stronger inhibitory effect of TMSD than the other positions. Nine genes were tested by fluorescence assay showing consistency with the simulation results. Low abundance of variants can be detected down to 0.1% with 3 S/N by employing the probe‐sink system. High orthogonality of the probe system allows for simultaneous detection of SNVs in multiple genes. TMSD has been widely used for SNV detection, however, the choice of mismatch location varies resulting in unstable and unsatisfied SNV specificity. This work for the first time provides theoretical and experimental evidence for the choice of mismatch location. Furthermore, TMSD as a powerful tool in dynamic DNA nanotechnology has been widely exploited to construct DNA computing, logic devices, and drug delivery. Fine‐tuning of TMSD kinetics by mismatch holds great potential for developing smart and versatile DNA nanosystems.

## 4 EXPERIMENTAL SECTION

### 4.1 Materials and reagents

All oligonucleotides were purchased from Sangon Biotech (shanghai) Co., Ltd. Functionalized strand were purified by HPLC and other unmodified oligonucleotides were purified by PAGE. The sequences were listed in Table S1 in the Supporting Information. The 10×TAE buffer (400 mm Tris, 200 nm CH_3_COOH, 20 mm EDTA, 50 mm Mg^2+^) was prepared and stored at 4 ℃. The λ exo was obtained from New England Biolabs (NEB). DNase/RNase‐free deionized water was used in all experiments.

### 4.2 oxDNA simulation

TMSD was simulated through US and FFS by using oxDNA, which is a coarse‐grained MD simulation software program. oxView was used to build TMSD structures. We can autonomously adjust the position and orientation of each base and the DNA nanostructures are more intuitively shown in a graphical way. The initial simulation files for oxDNA, “prova.top” and “prova.dat,” are both generated from oxView. After the initial simulation files were prepared, we set the hydrogen bonding strength for the specific mismatch base pair. The line in code, “HYDR_A_T” and “HYDR_C_G,” are positive number representing normal right base pairs’ hydrogen bonding strength, a code “HYDR_G_G = −5.00″ was written in the file specifying base dependencies. This indicates the mismatch base pair G‐G cannot form normal hydrogen bonds. We firstly simulated TMSD by US under the “VMMC” type, PM system and four MM systems used different order parameters corresponding to weight settings (Tables S3–S7 in the Supporting Information). And free energies were calculated from the sample data. Then we used python scripts to run FFS in oxDNA because FFS is a process of repeated TMSD and possibilities of passing through each set interface were recorded (Table S8 in the Supporting Information). And we could compare the effects of mismatches at different positions on TMSD.

### 4.3 Characterization of the products of toehold‐mediated strand displacement products by polyacrylamide gel electrophoresis

The products of TMSD reactions were verified by native 10% PAGE (29:1 acrylamide/bisacrylamide). 0.5×TBE buffer (44.5 mm Tris, 44.5 mm Boric acid, 1 mm EDTA, pH 8.0) was used in PAGE. Each sample (5 μL, 100 nm) was mixed with 6×loading buffer (2 μL), then added in the gel hole of electrophoresis. All samples were run at 120V for 50 min. After 10 min of staining in SYBR GOLD (Invitrogen) dissolved in 0.5×TBE buffer, the gel was photographed with a gel imaging system (Tanon‐2500BR).

### 4.4 Real‐time detection of fluorescence

DNA probes were prepared by mixing the corresponding single strands with equal concentrations (1 μm) in Mg^2+^‐containing 1×TAE buffer (40 mm Tris, 20 mm Glacial acetic acid, 2 mm EDTA, 5 mm Mg^2+^) in PCR tubes (20 μL). The initial reaction systems were annealed in a PCR thermal cycler from 90 to 37 ℃ at a slow rate. Then target strand was added and fluorescence was recorded immediately in a real‐time fluorescence quantitative PCR cycler (Rotor‐Gene Q, Qiagen, Germany) at 25 ℃ using a gain of 8 and time interval of 5 s.

### 4.5 Single nucleotide variant detection in plasmid clones

The plasmids were constructed with *S gene* in *SRAS‐CoV‐2* and *EGFR gene* in cell genome, including mutant type and wild type. To a PCR tube (200 μL), forward primer (2.5 μL, 10 μm), reverse primer (2.5 μL, 10 μm), plasmid (1 μL, 1 ng μL^−1^), Q5 High‐Fidelity 2X Master Mix (25 μL) were added and the total volume was brought up to 50 μL by ddH2O. PCR procedure (98 ℃ for 10 s, 55 ℃ for 30 s, 72 ℃ for 30 s, 28 cycles) was performed in a PCR thermal cycler. After the PCR amplification, λ Exo (1 μL, 250U) is added to degrade the single strand containing 5′‐PO_4_ in the duplex products for 30 min at 37 ℃. Then mutant type and wild type were combined with corresponding prepared DNA probes (100 nm) to detect in real‐time fluorescence quantification.

## CONFLICT OF INTEREST

The authors declare no competing interests.

## Supporting information



SUPPORTING INFORMATION

## References

[1] R. Sachidanandam , D. Weissman , S. C. Schmidt , J. M. Kakol , L. D. Stein , G. Marth , S. Sherry , J. C. Mullikin , B. J. Mortimore , D. L. Willey , S. E. Hunt , C. G. Cole , P. C. Coggill , C. M. Rice , Z. Ning , J. Rogers , D. R. Bentley , P.‐Y. Kwok , E. R. Mardis , R. T. Yeh , B. Schultz , L. Cook , R. Davenport , M. Dante , L. Fulton , L. Hillier , R. H. Waterston , J. D. McPherson , B. Gilman , S. Schaffner , et al., Nature 2001, 409, 928. 11237013 10.1038/35057149

[2] S. Kim , A. J. A. R. o. B. E. Misra , Annu. Rev. Biomed. Eng. 2007, 9, 289. 17391067 10.1146/annurev.bioeng.9.060906.152037

[3] S. X. Chen , D. Y. Zhang , G. Seelig , Nat. Chem. 2013, 5, 782. 23965681 10.1038/nchem.1713 PMC3844531

[4] M. Russo , G. Crisafulli , A. Sogari , N. M. Reilly , S. Arena , S. Lamba , A. Bartolini , V. Amodio , A. Magrì , L. Novara , I. Sarotto , Z. D. Nagel , C. G. Piett , A. Amatu , A. Sartore‐Bianchi , S. Siena , A. Bertotti , L. Trusolino , M. Corigliano , M. Gherardi , M. C. Lagomarsino , F. D. Nicolantonio , A. Bardelli , Science 2019, 366, 1473. 31699882 10.1126/science.aav4474

[5] B. Korber , W. M. Fischer , S. Gnanakaran , H. Yoon , J. Theiler , W. Abfalterer , N. Hengartner , E. E. Giorgi , T. Bhattacharya , B. Foley , K. M. Hastie , M. D. Parker , D. G. Partridge , C. M. Evans , T. M. Freeman , T. I. de Silva , A. Angyal , R. L. Brown , L. Carrilero , L. R. Green , D. C. Groves , K. J. Johnson , A. J. Keeley , B. B. Lindsey , P. J. Parsons , M. Raza , S. Rowland‐Jones , N. Smith , R. M. Tucker , D. Wang , et al., Cell 2020, 182, 812. 32697968 10.1016/j.cell.2020.06.043 PMC7332439

[6] Y. Li , G. A. Wang , S. D. Mason , X. Yang , Z. Yu , Y. Tang , F. Li , Chem. Sci. 2018, 9, 6434. 30310573 10.1039/c8sc02761g PMC6115701

[7] a) L. Li , X. Xiao , J. Ge , M. Han , X. Zhou , L. Wang , X. Su , C. Yu , ACS Sens. 2017, 2, 419; 28723215 10.1021/acssensors.7b00005

[8] J. Kong , J. Zhu , U. F. Keyser , Chem. Commun. 2017, 53, 436. 10.1039/c6cc08621g 27965988

[9] L. Li, Y. Yu, C. Wang, Q. Han, X. Su, Anal. Chem. 2019, 91, 11122. 31402644 10.1021/acs.analchem.9b01766

[10] M. Cui , H. Feng , D. Guo , D. Wang , B. Zheng , Sens. Actuators B 2017, 253, 731.

[11] S. Shin , B. Y. Won , C. Jung , S. C. Shin , D. Y. Cho , S. S. Lee , H. G. Park , Chem. Commun. 2011, 47, 6611. 10.1039/c1cc11476j 21573274

[12] a) X. Wu , J. Wu , J. Dai , B. Chen , Z. Chen , S. Wang , F. Wu , X. Lou , F. Xia , Natl. Sci. Rev. 2021, 8, nwaa306; 34691667 10.1093/nsr/nwaa306 PMC8288165

[13] W. Tang , W. Zhong , Y. Tan , G. A. Wang , F. Li , Y. Liu , Top. Curr. Chem. 2020, 378, 10. 10.1007/s41061-019-0274-z 31894426

[14] a) L. Li , X. Xiao , J. Ge , M. Han , X. Zhou , L. Wang , X. Su , C. J. A. S. Yu , ACS Sens. 2017, 2, 419; 28723215 10.1021/acssensors.7b00005

[15] a) S. X. Chen , G. Seelig , J. Am. Chem. Soc. 2016, 138, 5076; 27010123 10.1021/jacs.6b00277

[16] a) R. R. F. Machinek , T. E. Ouldridge , N. E. C. Haley , J. Bath , A. J. Turberfield , Nat. Commun. 2014, 5, 5324; 25382214 10.1038/ncomms6324

[17] Y. Tan , X. Zhang , W. Tang , W. Zhong , J. Fan , D. Guo , X. Wu , Y. Liu , Anal. Chim. Acta 2021, 1145, 3. 33453878 10.1016/j.aca.2020.12.023

[18] J. S. Wang , D. Y. Zhang , Nat. Chem. 2015, 7, 545. 26100802 10.1038/nchem.2266 PMC4479422

[19] a) K. Zhang , R. Deng , H. Gao , X. Teng , J. Li , Chem. Soc. Rev. 2020, 49, 1932; 32108196 10.1039/c9cs00438f

[20] M. E. Fornace , N. J. Porubsky , N. A. Pierce , ACS Synth. Biol. 2020, 9, 2665. 32910644 10.1021/acssynbio.9b00523

[21] G. Park , M. K. Cho , Y. Jung , J. Chem. Theory Comput. 2021, 17, 1308. 33570937 10.1021/acs.jctc.0c01116

[22] E. Poppleton , J. Bohlin , M. Matthies , S. Sharma , F. Zhang , P. Šulc , Nucleic Acids Res. 2020, 48, e72. 32449920 10.1093/nar/gkaa417 PMC7337935

[23] a) N. Srinivas , T. E. Ouldridge , P. Šulc , J. M. Schaeffer , B. Yurke , A. A. Louis , J. P. K. Doye , E. Winfree , Nucleic Acids Res. 2013, 41, 10641; 24019238 10.1093/nar/gkt801 PMC3905871

[24] F. Hong , J. S. Schreck , P. Šulc , Nucleic Acids Res. 2020, 48, 10726. 33045749 10.1093/nar/gkaa854 PMC7641764

[25] Z. Zhang , I. M. Hsing , Anal. Chem. 2017, 89, 12466. 29069899 10.1021/acs.analchem.7b03564

[26] a) W. Zhong , W. Tang , J. Fan , J. Zhang , X. Zhou , Y. Liu , Chem. Commun. 2018, 54, 1311; 10.1039/c7cc07733e 29177325

[27] K. Y. Su , J. S. Tseng , K. M. Liao , T. Y. Yang , K. C. Chen , K. H. Hsu , P. C. Yang , S. L. Yu , G. C. Chang , PloS ONE 2018, 13, e0207001. 30444875 10.1371/journal.pone.0207001 PMC6239293

[28] W. Li , T. Qiu , L. Guo , Y. Ling , Y. Gao , J. Ying , J. He , Cancer Lett. 2018, 423, 9. 29524556 10.1016/j.canlet.2018.03.005

[29] T. L. Dao , V. T. Hoang , P. Colson , J.‐C. Lagier , M. Million , D. Raoult , A. Levasseur , P. Gautret , J. Clin. Med. 2021, 10, 2635. 34203844 10.3390/jcm10122635 PMC8232800

[30] L. Zhang , Y. Wang , Y. Guo , H. Chen , W. Yu , Z. Zhang , G. Xie , Anal. Chim. Acta 2021, 1166, 338545. 34023002 10.1016/j.aca.2021.338545

[31] J. A. de Cena , J. Zhang , D. Deng , N. Damé‐Teixeira , T. Do , Front. Cell. Infect. Microbiol. 2021, 11, 689197. 34136418 10.3389/fcimb.2021.689197 PMC8201079

[32] a) L. A. Loeb , K. R. Loeb , J. P. Anderson , Proc. Natl. Acad. Sci. U. S. A. 2003, 100, 776; 12552134 10.1073/pnas.0334858100 PMC298677

