# Supplementary material for: Molecular dynamics simulation‐guided toehold mediated strand displacement probe for single‐nucleotide variants detection
Source: Exploration (Beijing). 2022 Jan 24;2(1):20210265. doi: 10.1002/EXP.20210265 (PMC10190925; doi:10.1002/EXP.20210265)
Supplement: Supplementary file 1 — SUPPORTING INFORMATION [file EXP2-2-20210265-s001.pdf]

Supporting Information

Molecular Dynamics Simulation-Guided Toehold Mediated Strand Displacement Probe for Single-Nucleotide Variants Detection

Linghao Zhang,[a] *Jing Chen,*[a] *Mengya He,*[a] *and Xin Su,*[a,*]

[a] College of Life Science and Technology, Beijing University of Chemical Technology, Beijing 100029, China.

[*] Corresponding author

Email: xinsu@mail.buct.edu.cn

Tel: +86-10-64421335

Content

Supporting Tables 1

Table S1. Sequence of the oligonucleotides in this work. 1

Table S2. △G by NUPACK analysis. 5

Table S3. Biasing weight W using in simulation of PM-TMSD system. 6

Table S4. Biasing weight W using in simulation of MM-TMSD (Toe4) system. 7

Table S5. Biasing weight W using in simulation of MM-TMSD (Toe7) system. 7

Table S6. Biasing weight W using in simulation of MM-TMSD (BM1) system. 8

Table S7. Biasing weight W using in simulation of MM-TMSD (BM11) system. 8

Table S8. Simulation data obtained from FFS simulations of PM-TMSD and MM-TMSD. 9

Supporting Figures 10

Supporting Tables

Table S1. Sequence of the oligonucleotides in this work.

Name	Sequence (5'-3')
Universal fluorescent strand and quencher strand	
FAM-20	FAM-ACACCTTTAACCCGTACCAT
HEX-20	HEX-ACACCTTTAACCCGTACCAT
ROX-20	ROX-ACACCTTTAACCCGTACCAT
CY5-20	CY5-ACACCTTTAACCCGTACCAT
Q1-22	AAGGAGCACAGGTTACCATAAG-BHQ1
Q3-22	AAGGAGCACAGGTTACCATAAG-BHQ3
Strands for testing the nine genes	
EGFR-L858R(SNV4)	ATGTCAAGATCACAGATTTTGGGCGGGC
EGFR-L858R(WT4)	ATGTCAAGATCACAGATTTTGGGCTGGC
F_L858R(4)	ATGGTACGGGTTAAAGGTGTATGTCAAGATCACAGATTTTG
Q_L858R(4)	GCCCGCCCAAAATCTGTGATCTTGACATCTTATGGTAACCTGTGCTCCTT
EGFR-L858R(SNV7)	TCAAGATCACAGATTTTGGGCGGGCCAA
EGFR-L858R(WT7)	TCAAGATCACAGATTTTGGGCTGGCCAA
F_L858R(7)	ATGGTACGGGTTAAAGGTGTTCAAGATCACAGATTTTGGGC
Q_L858R(7)	TTGGCCCGCCCAAAATCTGTGATCTTGACTTATGGTAACCTGTGCTCCTT
EGFR-L858R(SNV1)	CAAGATCACAGATTTTGGGCGGGCCAAA
EGFR-L858R(WT1)	CAAGATCACAGATTTTGGGCTGGCCAAA
F_L858R(1)	ATGGTACGGGTTAAAGGTGTCAAGATCACAGATTTTGGGCG
Q_L858R(1)	TTTGGCCCGCCCAAAATCTGTGATCTTGCTTATGGTAACCTGTGCTCCTT
EGFR-L858R(SNV11)	GATTTTGGGCGGGCCAAACTGCTGGGTG
EGFR-L858R(WT11)	GATTTTGGGCTGGCCAAACTGCTGGGTG
F_L858R(11)	ATGGTACGGGTTAAAGGTGTGATTTTGGGCGGGCCAAACTG
Q_L858R(11)	CACCCAGCAGTTTGGCCCGCCCAAAATCCTTATGGTAACCTGTGCTCCTT
NRAS-G12C(SNV4)	TACAAACTGGTGGTGGTTGGAGCATGTG
NRAS-G12C(WT4)	TACAAACTGGTGGTGGTTGGAGCAGGTG
F_G12C(4)	ATGGTACGGGTTAAAGGTGTTACAAACTGGTGGTGGTTGGA
Q_G12C(4)	CACATGCTCCAACCACCACCAGTTTGTACTTATGGTAACCTGTGCTCCTT
NRAS-G12C(SNV7)	AAACTGGTGGTGGTTGGAGCATGTGGTG
NRAS-G12C(WT7)	AAACTGGTGGTGGTTGGAGCAGGTGGTG
F_G12C(7)	ATGGTACGGGTTAAAGGTGTAAACTGGTGGTGGTTGGAGCA
Q_G12C(7)	CACCACATGCTCCAACCACCACCAGTTTCTTATGGTAACCTGTGCTCCTT
NRAS-G12C(SNV1)	AACTGGTGGTGGTTGGAGCATGTGGTGT
NRAS-G12C(WT1)	AACTGGTGGTGGTTGGAGCAGGTGGTGT
F_G12C(1)	ATGGTACGGGTTAAAGGTGTAACTGGTGGTGGTTGGAGCAT
Q_G12C(1)	ACACCACATGCTCCAACCACCACCAGTTCTTATGGTAACCTGTGCTCCTT
NRAS-G12C(SNV11)	GGTTGGAGCATGTGGTGTTGGGAAAAGC
NRAS-G12C(WT11)	GGTTGGAGCAGGTGGTGTTGGGAAAAGC
F_G12C(11)	ATGGTACGGGTTAAAGGTGTGGTTGGAGCATGTGGTGTTGG

Q_G12C(11)	GCTTTTCCCAACACCACATGCTCCAACCCTTATGGTAACCTGTGCTCCTT
N501Y(SNV4)	CAAAGCTACGGTTTCCAACCGACCTACG
N501Y(WT4)	CAAAGCTACGGTTTCCAACCGACCAACG
F_N501Y(4)	ATGGTACGGGTTAAAGGTGTCAAAGCTACGGTTTCCAACCG
Q_N501Y(4)	CGTAGGTCGGTTGGAAACCGTAGCTTTGCTTATGGTAACCTGTGCTCCTT
N501Y(SNV7)	AGCTACGGTTTCCAACCGACCTACGGTG
N501Y(WT7)	AGCTACGGTTTCCAACCGACCAACGGTG
F_N501Y(7)	ATGGTACGGGTTAAAGGTGTAGCTACGGTTTCCAACCGACC
Q_N501Y(7)	CACCGTAGGTCGGTTGGAAACCGTAGCTCTTATGGTAACCTGTGCTCCTT
N501Y(SNV1)	GCTACGGTTTCCAACCGACCTACGGTGT
N501Y(WT1)	GCTACGGTTTCCAACCGACCAACGGTGT
F_N501Y(1)	ATGGTACGGGTTAAAGGTGTGCTACGGTTTCCAACCGACCT
Q_N501Y(1)	ACACCGTAGGTCGGTTGGAAACCGTAGCCTTATGGTAACCTGTGCTCCTT
N501Y(SNV11)	CCAACCGACCTACGGTGTTGGTTACCAG
N501Y(WT11)	CCAACCGACCAACGGTGTTGGTTACCAG
F_N501Y(11)	ATGGTACGGGTTAAAGGTGTCCAACCGACCTACGGTGTTGG
Q_N501Y(11)	CTGGTAACCAACACCGTAGGTCGGTTGGCTTATGGTAACCTGTGCTCCTT
D614G(SNV4)	ACCAGGTTGCGGTGCTGTATCAAGGCGT
D614G(WT4)	ACCAGGTTGCGGTGCTGTATCAAGACGT
F_D614G(4)	ATGGTACGGGTTAAAGGTGTACCAGGTTGCGGTGCTGTATC
Q_D614G(4)	ACGCCTTGATACAGCACCGCAACCTGGTCTTATGGTAACCTGTGCTCCTT
D614G(SNV7)	AGGTTGCGGTGCTGTATCAAGGCGTTAA
D614G(WT7)	AGGTTGCGGTGCTGTATCAAGACGTTAA
F_D614G(7)	ATGGTACGGGTTAAAGGTGTAGGTTGCGGTGCTGTATCAAG
Q_D614G(7)	TTAACGCCTTGATACAGCACCGCAACCTCTTATGGTAACCTGTGCTCCTT
D614G(SNV1)	GGTTGCGGTGCTGTATCAAGGCGTTAAC
D614G(WT1)	GGTTGCGGTGCTGTATCAAGACGTTAAC
F_D614G(1)	ATGGTACGGGTTAAAGGTGTGGTTGCGGTGCTGTATCAAGG
Q_D614G(1)	GTTAACGCCTTGATACAGCACCGCAACCCTTATGGTAACCTGTGCTCCTT
D614G(SNV11)	CTGTATCAAGGCGTTAACTGCACCGAAG
D614G(WT11)	CTGTATCAAGACGTTAACTGCACCGAAG
F_D614G(11)	ATGGTACGGGTTAAAGGTGTCTGTATCAAGGCGTTAACTGC
Q_D614G(11)	CTTCGGTGCAGTTAACGCCTTGATACAGCTTATGGTAACCTGTGCTCCTT
PIKC3CA-H1047R(SNV4)	TCATGAAACAAATGAATGATGCACGTCA
PIKC3CA-H1047R(WT4)	TCATGAAACAAATGAATGATGCACATCA
F_H1047R(4)	ATGGTACGGGTTAAAGGTGTTCATGAAACAAATGAATGATG
Q_H1047R(4)	TGACGTGCATCATTCATTTGTTTCATGACTTATGGTAACCTGTGCTCCTT
PIKC3CA-H1047R(SNV7)	TGAAACAAATGAATGATGCACGTCATGG
PIKC3CA-H1047R(WT7)	TGAAACAAATGAATGATGCACATCATGG
F_H1047R(7)	ATGGTACGGGTTAAAGGTGTTGAAACAAATGAATGATGCAC
Q_H1047R(7)	CCATGACGTGCATCATTCATTTGTTTCACTTATGGTAACCTGTGCTCCTT
PIKC3CA-H1047R(SNV1)	GAAACAAATGAATGATGCACGTCATGGT
PIKC3CA-H1047R(WT1)	GAAACAAATGAATGATGCACATCATGGT
F_H1047R(1)	ATGGTACGGGTTAAAGGTGTGAAACAAATGAATGATGCACG

Q_H1047R(1)	ACCATGACGTGCATCATTCATTTGTTTCCTTATGGTAACCTGTGCTCCTT
PIKC3CA-H1047R(SNV11)	AATGATGCACATCATGGTGGCTGGACAA
PIKC3CA-H1047R(WT11)	AATGATGCACGTCATGGTGGCTGGACAA
F_H1047R(11)	ATGGTACGGGTTAAAGGTGTAATGATGCACATCATGGTGGC
Q_H1047R(11)	TTGTCCAGCCACCATGATGTGCATCATTCTTATGGTAACCTGTGCTCCTT
KRAS-G12R(SNV4)	TATAAACTTGTGGTAGTTGGAGCTCGTG
KRAS-G12R(WT4)	TATAAACTTGTGGTAGTTGGAGCTGGTG
F_G12R(4)	ATGGTACGGGTTAAAGGTGTTATAAACTTGTGGTAGTTGGA
Q_G12R(4)	CACGAGCTCCAACTACCACAAGTTTATACTTATGGTAACCTGTGCTCCTT
KRAS-G12R(SNV7)	AAACTTGTGGTAGTTGGAGCTCGTGGCG
KRAS-G12R(WT7)	AAACTTGTGGTAGTTGGAGCTGGTGGCG
F_G12R(7)	ATGGTACGGGTTAAAGGTGTAAACTTGTGGTAGTTGGAGCT
Q_G12R(7)	CGCCACGAGCTCCAACTACCACAAGTTTCTTATGGTAACCTGTGCTCCTT
KRAS-G12R(SNV1)	AACTTGTGGTAGTTGGAGCTCGTGGCGT
KRAS-G12R(WT1)	AACTTGTGGTAGTTGGAGCTGGTGGCGT
F_G12R(1)	ATGGTACGGGTTAAAGGTGTAACTTGTGGTAGTTGGAGCTC
Q_G12R(1)	ACGCCACGAGCTCCAACTACCACAAGTTCTTATGGTAACCTGTGCTCCTT
KRAS-G12R(SNV11)	AGTTGGAGCTCGTGGCGTAGGCAAGAGT
KRAS-G12R(WT11)	AGTTGGAGCTGGTGGCGTAGGCAAGAGT
F_G12R(11)	ATGGTACGGGTTAAAGGTGTAGTTGGAGCTCGTGGCGTAGG
Q_G12R(11)	ACTCTTGCCTACGCCACGAGCTCCAACTCTTATGGTAACCTGTGCTCCTT
STK11-F354L(SNV4)	CGCGGACGAGGACGAGGACCTCTTGGAC
STK11-F354L(WT4)	CGCGGACGAGGACGAGGACCTCTTCGAC
F_F354L(4)	ATGGTACGGGTTAAAGGTGTCGCGGACGAGGACGAGGACCT
Q_F354L(4)	GTCCAAGAGGTCCTCGTCCTCGTCCGCGCTTATGGTAACCTGTGCTCCTT
STK11-F354L(SNV7)	GGACGAGGACGAGGACCTCTTGGACATC
STK11-F354L(WT7)	GGACGAGGACGAGGACCTCTTCGACATC
F_F354L(7)	ATGGTACGGGTTAAAGGTGTGGACGAGGACGAGGACCTCTT
Q_F354L(7)	GATGTCCAAGAGGTCCTCGTCCTCGTCCCTTATGGTAACCTGTGCTCCTT
STK11-F354L(SNV1)	GACGAGGACGAGGACCTCTTGGACATCG
STK11-F354L(WT1)	GACGAGGACGAGGACCTCTTCGACATCG
F_F354L(1)	ATGGTACGGGTTAAAGGTGTGACGAGGACGAGGACCTCTTG
Q_F354L(1)	CGATGTCCAAGAGGTCCTCGTCCTCGTCCTTATGGTAACCTGTGCTCCTT
STK11-F354L(SNV11)	AGGACCTCTTGGACATCGAGGATGACAT
STK11-F354L(WT11)	AGGACCTCTTCGACATCGAGGATGACAT
F_F354L(11)	ATGGTACGGGTTAAAGGTGTAGGACCTCTTGGACATCGAGG
Q_F354L(11)	ATGTCATCCTCGATGTCCAAGAGGTCCTCTTATGGTAACCTGTGCTCCTT
TP53-Y220C(SNV4)	TTCGACATAGTGTGGTGGTGCCCTGTGA
TP53-Y220C(WT4)	TTCGACATAGTGTGGTGGTGCCCTATGA
F_Y220C(4)	ATGGTACGGGTTAAAGGTGTTTCGACATAGTGTGGTGGTGC
Q_Y220C(4)	TCACAGGGCACCACCACACTATGTCGAACTTATGGTAACCTGTGCTCCTT
TP53-Y220C(SNV7)	GACATAGTGTGGTGGTGCCCTGTGAGCC
TP53-Y220C(WT7)	GACATAGTGTGGTGGTGCCCTATGAGCC
F_Y220C(7)	ATGGTACGGGTTAAAGGTGTGACATAGTGTGGTGGTGCCCT

Q_Y220C(7)	GGCTCACAGGGCACCACCACACTATGTCCTTATGGTAACCTGTGCTCCTT
TP53-Y220C(SNV1)	ACATAGTGTGGTGGTGCCCTGTGAGCCG
TP53-Y220C(WT1)	ACATAGTGTGGTGGTGCCCTATGAGCCG
F_Y220C(1)	ATGGTACGGGTTAAAGGTGTACATAGTGTGGTGGTGCCCTG
Q_Y220C(1)	CGGCTCACAGGGCACCACCACACTATGTCTTATGGTAACCTGTGCTCCTT
TP53-Y220C(SNV11)	GTGGTGCCCTGTGAGCCGCCTGAGGTCT
TP53-Y220C(WT11)	GTGGTGCCCTATGAGCCGCCTGAGGTCT
F_Y220C(11)	ATGGTACGGGTTAAAGGTGTGTGGTGCCCTGTGAGCCGCCT
Q_Y220C(11)	AGACCTCAGGCGGCTCACAGGGCACCACCTTATGGTAACCTGTGCTCCTT
EGFR-T790M(SNV4)	TCACCTCCACCGTGCAGCTCATCATGCA
EGFR-T790M(WT4)	TCACCTCCACCGTGCAGCTCATCACGCA
F_T790M(4)	ATGGTACGGGTTAAAGGTGTTCACCTCCACCGTGCAGCTCA
Q_T790M(4)	TGCATGATGAGCTGCACGGTGGAGGTGACTTATGGTAACCTGTGCTCCTT
EGFR-T790M(SNV7)	CCTCCACCGTGCAGCTCATCATGCAGCT
EGFR-T790M(WT7)	CCTCCACCGTGCAGCTCATCACGCAGCT
F_T790M(7)	ATGGTACGGGTTAAAGGTGTCCTCCACCGTGCAGCTCATCA
Q_T790M(7)	AGCTGCATGATGAGCTGCACGGTGGAGGCTTATGGTAACCTGTGCTCCTT
EGFR-T790M(SNV1)	CTCCACCGTGCAGCTCATCATGCAGCTC
EGFR-T790M(WT1)	CTCCACCGTGCAGCTCATCACGCAGCTC
F_T790M(1)	ATGGTACGGGTTAAAGGTGTCTCCACCGTGCAGCTCATCAT
Q_T790M(1)	GAGCTGCATGATGAGCTGCACGGTGGAGCTTATGGTAACCTGTGCTCCTT
EGFR-T790M(SNV11)	CAGCTCATCATGCAGCTCATGCCCTTCG
EGFR-T790M(WT11)	CAGCTCATCACGCAGCTCATGCCCTTCG
F_T790M(11)	ATGGTACGGGTTAAAGGTGTCAGCTCATCATGCAGCTCATG
Q_T790M(11)	CGAAGGGCATGAGCTGCATGATGAGCTGCTTATGGTAACCTGTGCTCCTT
Strands for probe-sink systems	
D614G-S1	AGGTTGCGGTGCTGTATCAAG
D614G-S2	TTAACGTCTTGATACAGCACCGCAACCT
L858R-S1	TCAAGATCACAGATTTTGGGC
L858R-S2	TTGGCCAGCCCAAAATCTGTGATCTTGA
T790M-S1	CCTCCACCGTGCAGCTCATCA
T790M-S2	AGCTGCGTGATGAGCTGCACGGTGGAGG
PCR Primers	
D614G-F.P	GGGCACCAACACCAGCAACC
D614G-R.P	P-CCACCGGAACTTCGGTGCAG
N501Y-F.P	ACTGCTATTTCCCGCTGCAA
N501Y-R.P	P-ACGGTACGGCTGGTAACCAA
EGFR-L858R-F.P	GGTGAAAACACCGCAGCATG
EGFR-L858R-R.P	P-TCTCTTCCGCACCCAGCAGT

Note: F_L858R(4), Q_L858R(4), FAM-20, and Q1-22 forms TWJ probe to detect EGFR-L858R(SNV4) and EGFR-L858R(WT4). In brackets, (4), (7), (1), and (11) are corresponding to Toe4, Toe7, BM1, and BM11. In universal strands, FAM-20, HEX-20, ROX-20, and CY5-20 are the same sequence fluorescent strands with different fluorophore for multiple detection. BHQ1 quenches FAM, HEX and ROX, BHQ3 quenches CY5.

Table S2. △G by NUPACK analysis.

	△G (kcal/mol)			
	Toe4	Toe7	BM1	BM11
PM	-14.33	-15.4	-14.58	-18
MM	-8.59	-8.86	-8.17	-11.77

△G of PM-TMSD and MM-TMSDs were predicted by NUPACK. △△G is the difference between $△G_{PM}$ and $△G_{MM}$.

Conditions and settings in NUPACK:
salinity (Na^{+}: 0.05M; Mg^{2+}: 0.005M);
temperature (25℃);
concentration (200 nM);
dangle treatment (Some).

US simulation

Table S3. Biasing weight W using in simulation of PM-TMSD system.

N_B \ N_A	0	1	2	3	4	5	6	7	8	9	…	17	18	19	…	25	26	27	28
0	0.1	1	1	1	1	1	1	1	1	1		1	1	1		1	1	1	1
1	0.1															2000	2000	200	
2	0.1															2000	200		
3	0.1															200			
4	0.1																		
5	0.1																		
6	0.1																		
7	0.1													2000					
8	0.1												2000	2000					
9	0.1											2000	2000	200					
10	0.1											2000	200						
11	0.1											200							
…																			
17	0.1									2000									
18	0.1								2000	2000									
19	0.1	3000	1200	400	200	20	5	2	2000	200									
20	0.1	3000	1200	400	200	20	5	2	200										
21	0.1	3000	1200	400	200	20	5	2											

N_A is the number of base pairs between invader strand and template strand, and N_B is the number of base pairs between incumbent strand and template strand. The design of W depends on whether a mismatch is present. $W_{MM}=10\times W_{PM}$.

Table S4. Biasing weight W using in simulation of MM-TMSD (Toe4) system.

N_B \ N_A	0	1	2	3	4	5	6	7	8	…	17	18	19	…	25	26	27
0	0.1	1	1	1	1	1	1	1	1		1	1	1		1	1	1
1	0.1														2000	200	
2	0.1														200		
3	0.1																
4	0.1																
5	0.1																
6	0.1												2000				
7	0.1											2000	2000				
8	0.1										2000	2000	200				
9	0.1										2000	200					
10	0.1										200						
…																	
17	0.1								2000								
18	0.1							2000	2000								
19	0.1	3000	1200	400	500	20	5	2000	200								
20	0.1	3000	1200	400	500	20	5	200									
21	0.1	3000	1200	400	500	20	5										

Table S5. Biasing weight W using in simulation of MM-TMSD (Toe7) system.

N_B \ N_A	0	1	2	3	4	5	6	7	8	…	17	18	19	…	25	26	27
0	0.1	1	1	1	1	1	1	1	1		1	1	1		1	1	1
1	0.1														20000	2000	
2	0.1														2000		
3	0.1																
4	0.1																
5	0.1																
6	0.1												20000				
7	0.1											20000	20000				
8	0.1										20000	20000	2000				
9	0.1										20000	2000					
10	0.1										2000						
…																	
17	0.1								20000								
18	0.1							20000	20000								
19	0.1	3000	1200	400	500	20	5	20000	2000								
20	0.1	3000	1200	400	500	20	5	2000									
21	0.1	3000	1200	400	500	20	5										

Table S6. Biasing weight W using in simulation of MM-TMSD (BM1) system.

N_B \ N_A	0	1	2	3	4	5	6	7	8	9	…	17	18	19	…	25	26	27
0	0.1	1	1	1	1	1	1	1	1	1		1	1	1		1	1	1
1	0.1															20000	2000	
2	0.1															2000		
3	0.1																	
4	0.1																	
5	0.1																	
6	0.1													20000				
7	0.1												20000	20000				
8	0.1											20000	20000	2000				
9	0.1											20000	2000					
10	0.1											2000						
…																		
16										20000								
17	0.1								20000	20000								
18	0.1								20000	2000								
19	0.1	3000	1200	400	200	20	5	2	2000									
20	0.1	3000	1200	400	200	20	5	2										
21	0.1	3000	1200	400	200	20	5	2										

Table S7. Biasing weight W using in simulation of MM-TMSD (BM11) system.

N_B \ N_A	0	1	2	3	4	5	6	7	8	9	…	17	18	19	…	25	26	27
0	0.1	1	1	1	1	1	1	1	1	1		1	1	1		1	1	1
1	0.1															20000	2000	
2	0.1															2000		
3	0.1																	
4	0.1																	
5	0.1																	
6	0.1													20000				
7	0.1												20000	20000				
8	0.1											2000	20000	2000				
9	0.1											2000	2000					
10	0.1											200						
…	0.1																	
16										2000								
17	0.1								2000	2000								
18	0.1								2000	200								
19	0.1	3000	1200	400	200	20	5	2	200									
20	0.1	3000	1200	400	200	20	5	2										
21	0.1	3000	1200	400	200	20	5	2										

Table S8. Simulation data obtained from FFS simulations of PM-TMSD and MM-TMSD.

	PM	Toe4 mismatch	Toe7 mismatch	BM1 mismatch	BM11 mismatch
1/flux	17859.54	17606.5	17560.41	17980.8	17005.78
			success/attempts		
Toe1	100/11051	100/61114	101/15704	101/30906	100/32862
Toe2	106/346	103/306	103/233	102/341	102/709
Toe3	106/158	106/157	105/155	106/146	105/142
Toe4	101/227		105/179	103/160	101/192
Toe5	101/336	101/456	101/213	104/203	105/179
Toe6	102/341	102/284	101/370	101/232	102/164
Toe7	100/406	100/515		102/273	102/649
BM1	100/120	100/448	100/2476		104/135
BM2	100/297	100/3542	100/3776	100/36724	100/1957
BM3	100/2145	100/2980	101/4121	100/16950	101/4366
BM4	100/172	100/524	102/294	100/7368	101/355
BM5	100/1013	100/1083	100/4240	100/1508	100/2289
BM6	100/1325	100/1249	100/1160	101/564	100/1400
BM7	101/683	101/861	100/3679	100/9424	100/1834
BM8	101/2793	100/3716	100/2901	100/3594	100/4697
BM9	100/1109	101/1145	102/825	100/5389	100/1710
BM10	101/2358	100/3338	101/3665	100/5159	100/2673
BM11	101/1532	100/1856	100/1216	100/1886	
BM12	100/1304	100/1624	101/1967	100/3696	100/51128
BM13	101/1033	100/1840	100/2326	100/1962	100/1132
BM14	101/1366	100/1266	101/1285	100/3098	100/2292
BM15	100/1766	101/2222	100/883	101/1631	102/340

“Attempts” represent the repeats of independent trial.
“Success” represents the statistics of success to completely displace one base.
Success probability is defined as the ratio of the success number to attempts number.
e.g. success probability $_{\text{(Toe1 in PM)}}$ = 100 / 11051 = 0.009049

Supporting Figures

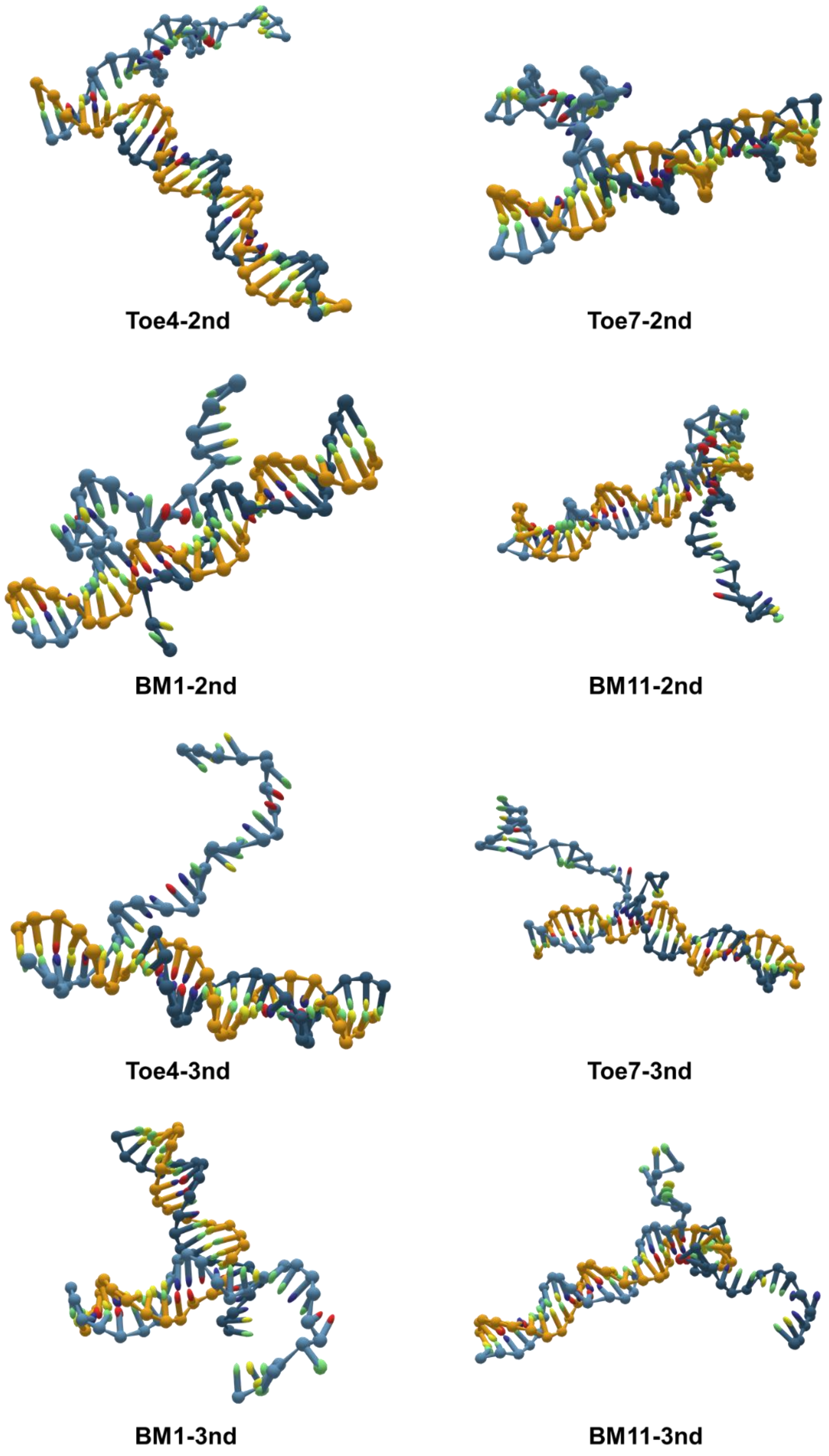

Figure S1. Conformations about base pairs formed after mismatch.

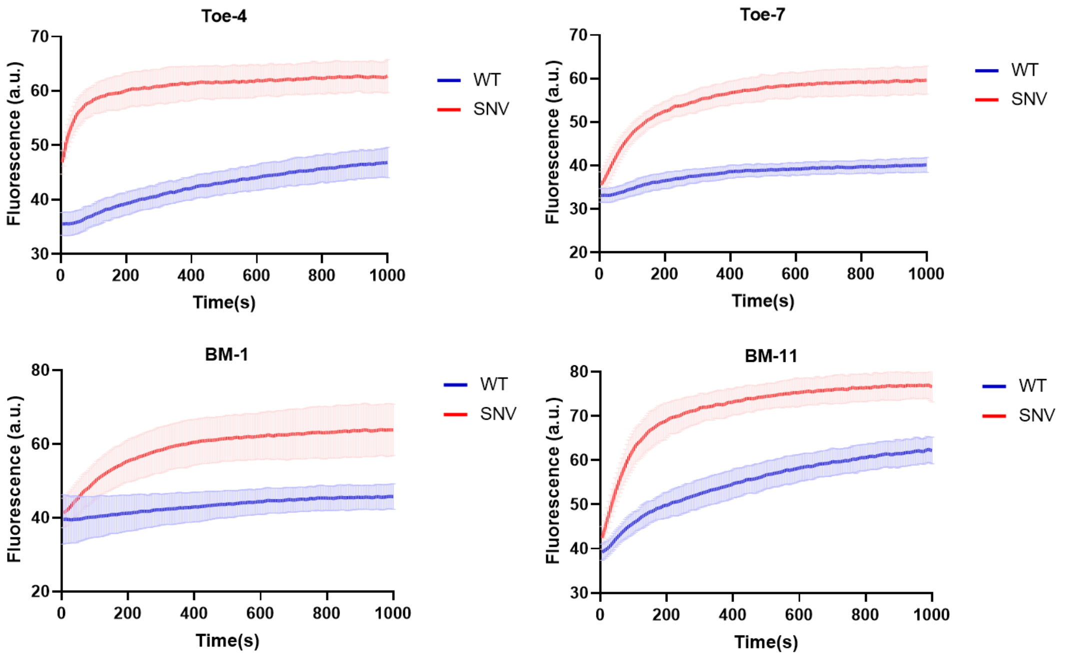

Figure S2. SNV detection of SARS-CoV-2-D614G by four kinds of probes.

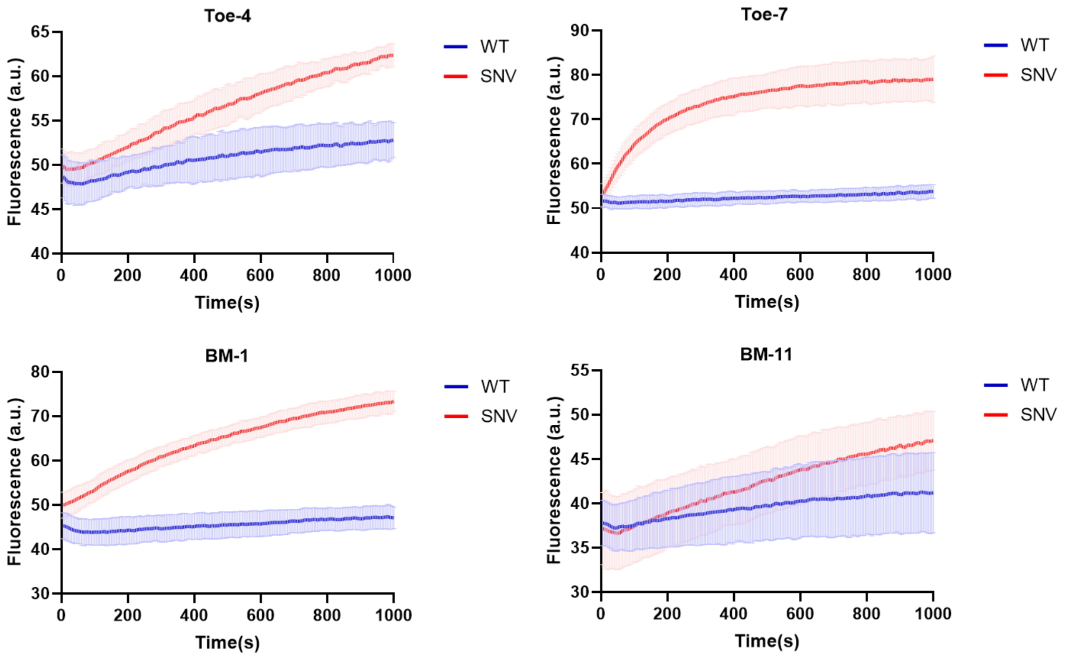

Figure S3. SNV detection of SARS-CoV-2-N501Y by four kinds of probes.

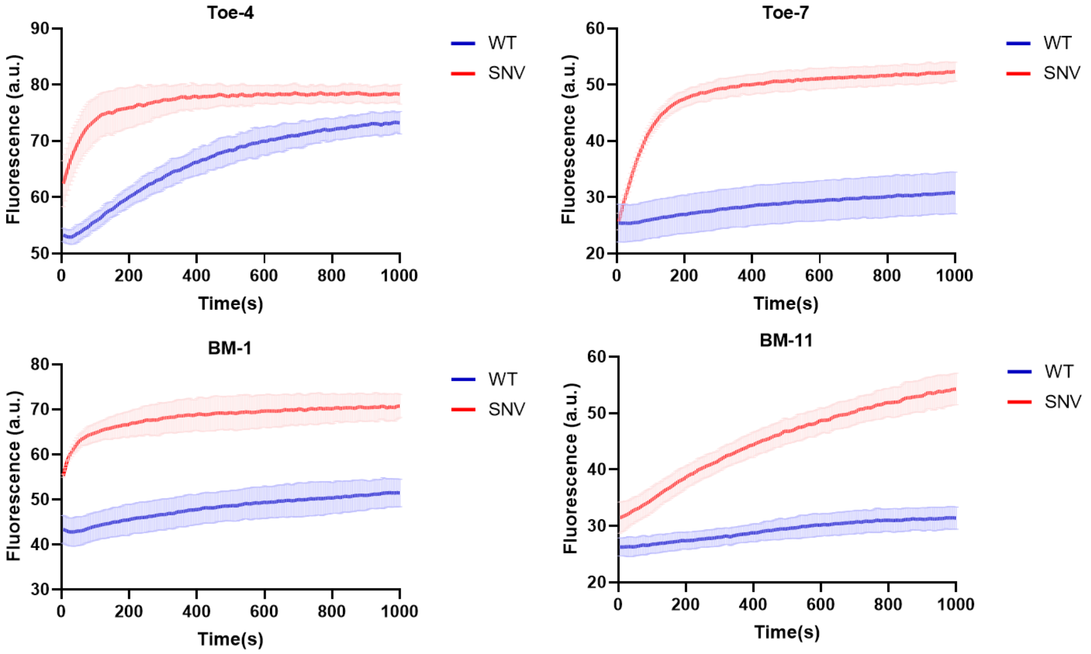

Figure S4. SNV detection of EGFR-L858R by four kinds of probes.

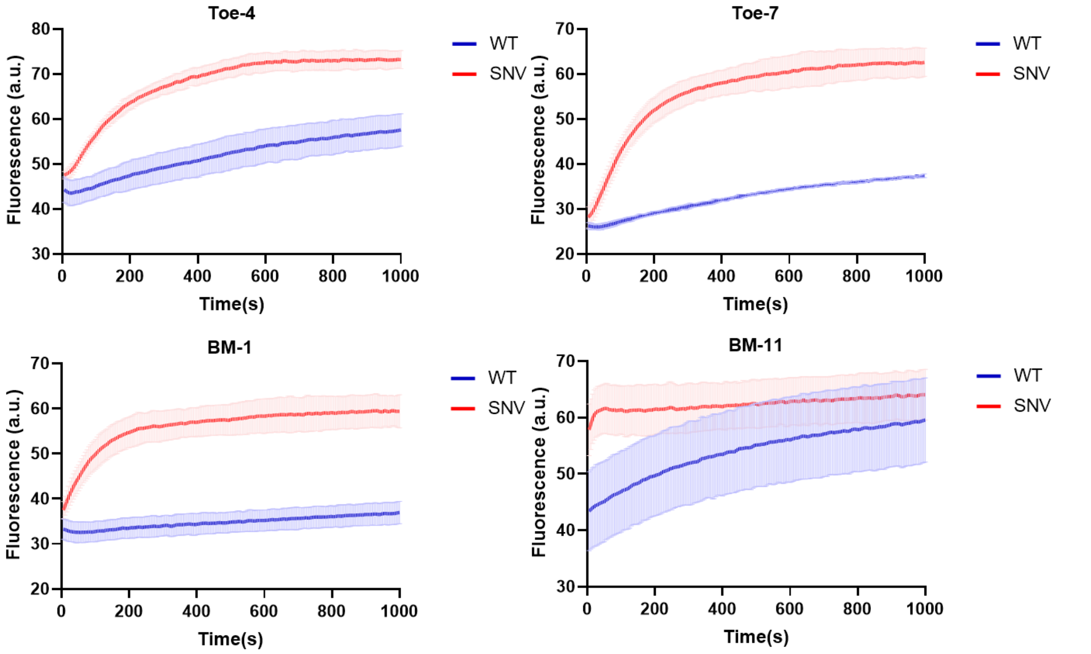

Figure S5. SNV detection of EGFR-T790M by four kinds of probes.

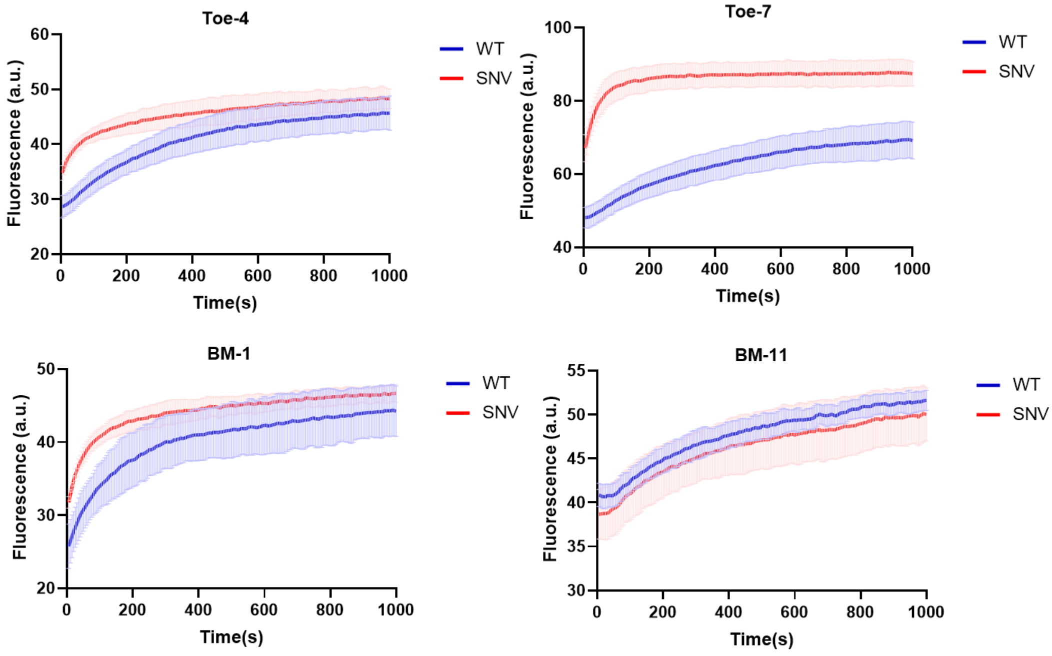

Figure S6. SNV detection of NRAS-G12C by four kinds of probes.

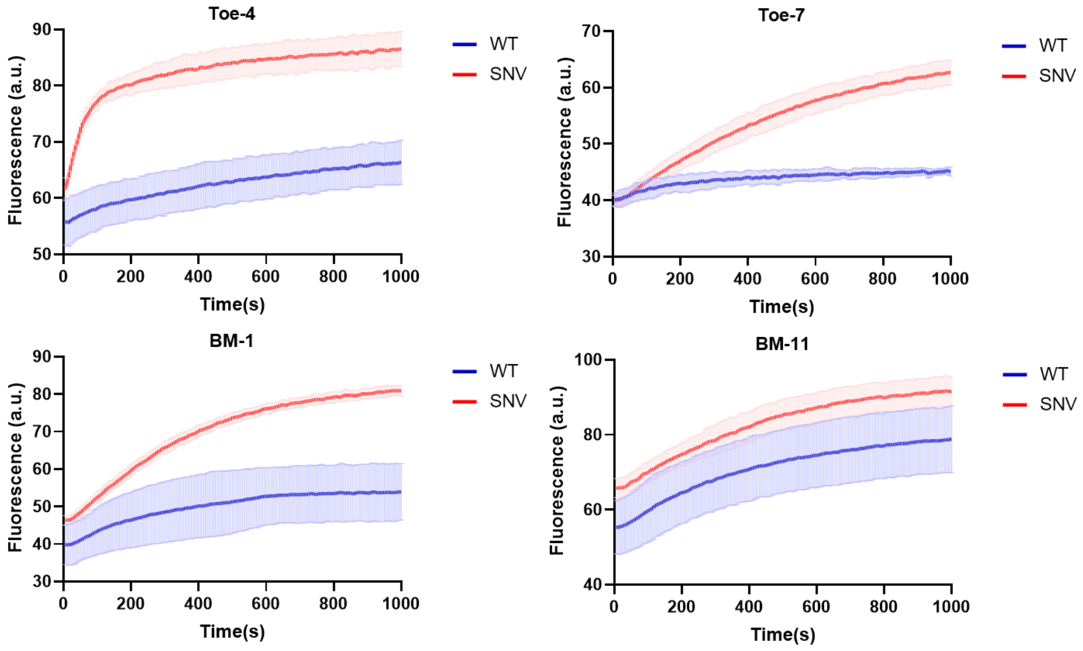

Figure S7. SNV detection of PIK3CA-H1047R by four kinds of probes.

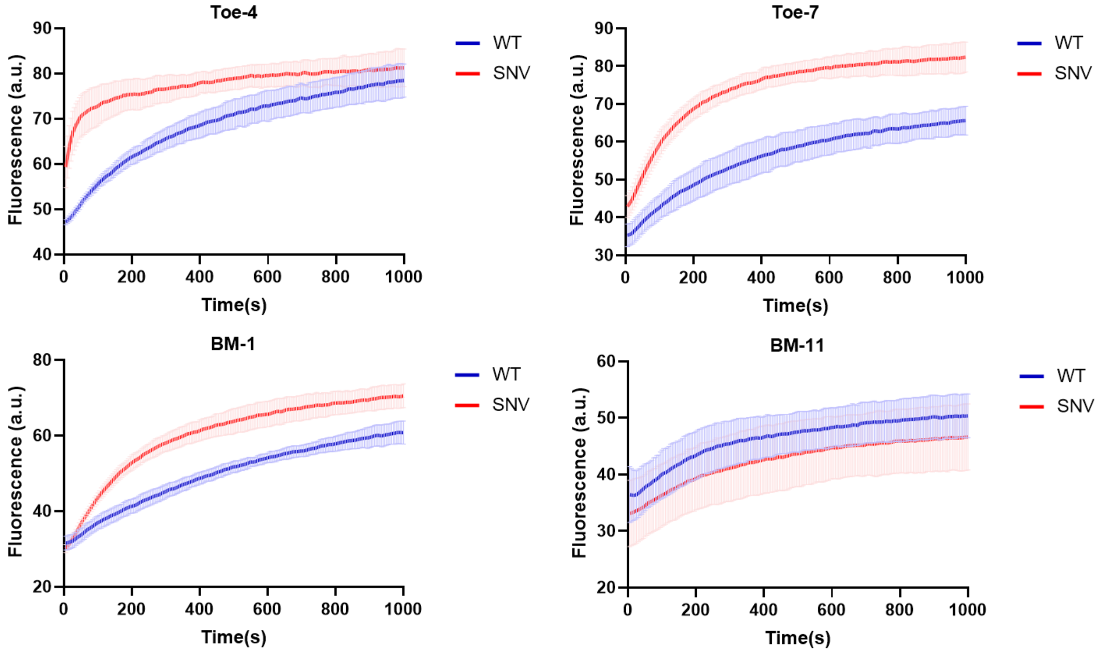

Figure S8. SNV detection of KRAS-G12R by four kinds of probes.

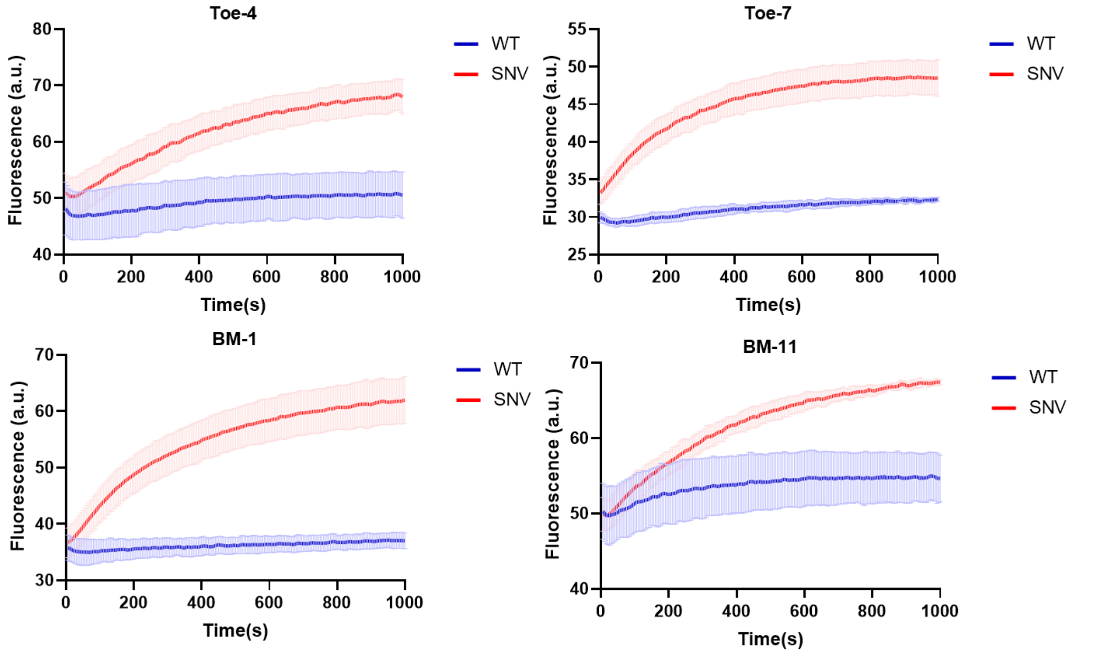

Figure S9. SNV detection of STK11-F354L by four kinds of probes.

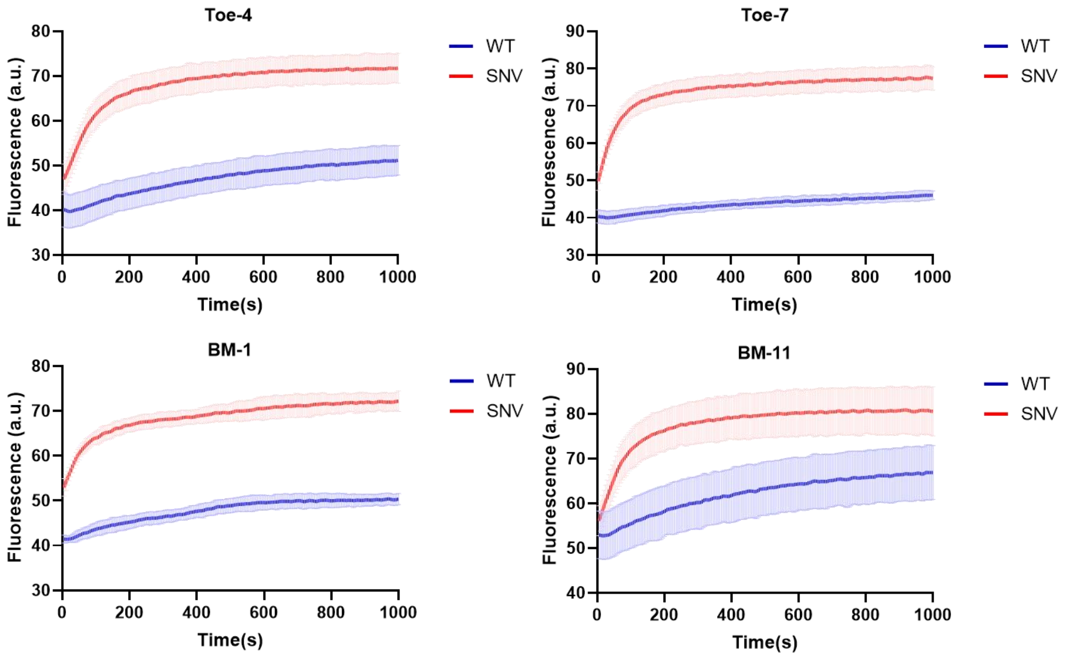

Figure S10. SNV detection of TP53-Y220C by four kinds of probes.

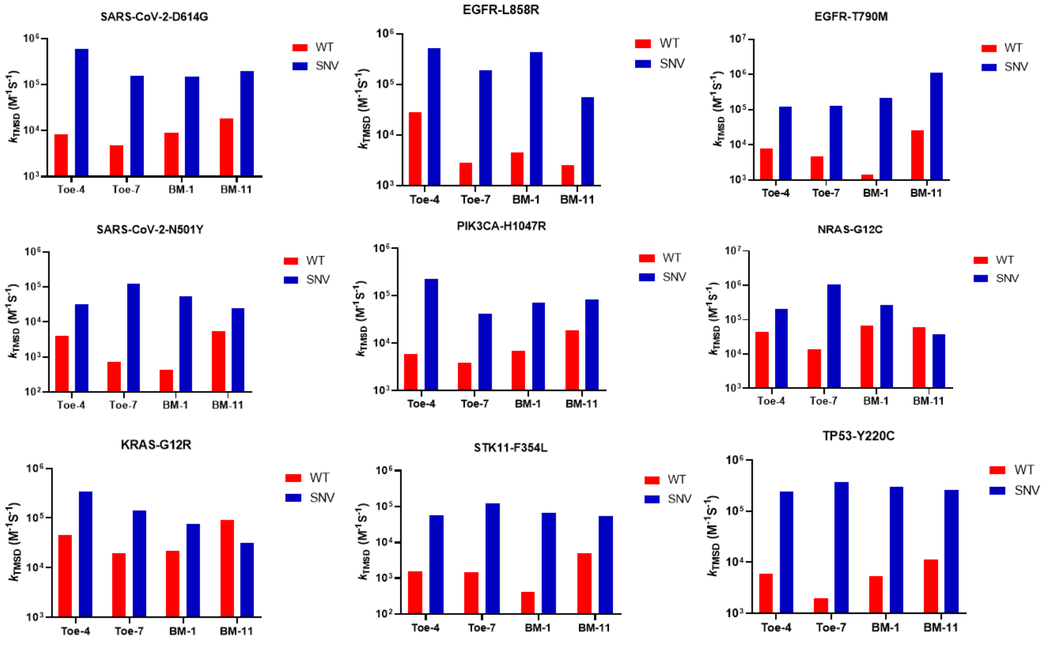

Figure S11. TMSD reaction constants. The second order kinetics equation was used to fit the experimental data in Figure S2-S10 to obtain TMSD reaction constants.

$$A + B \xrightarrow{k_{\mathrm{TMSD}}} C + D$$

d[D]/dt = k×[A]×[B]

[A] and [B] represent the concentrations of invader and template strand which are both 100 nM, [C] and [D] are the concentrations of quencher and fluorophore labeled strands, respectively. [D] was obtained from the fluorescence curve by the equation [D] = $(F-F_{initial})/(F_{final}-F_{initial})$ where F is

the fluorescence at a certain time, F_{final} and $F_{initial}$ represent the fluorescence intensity of the prepared ‘F’ structure and ‘TWJ’ structure (Figure 2A), respectively.

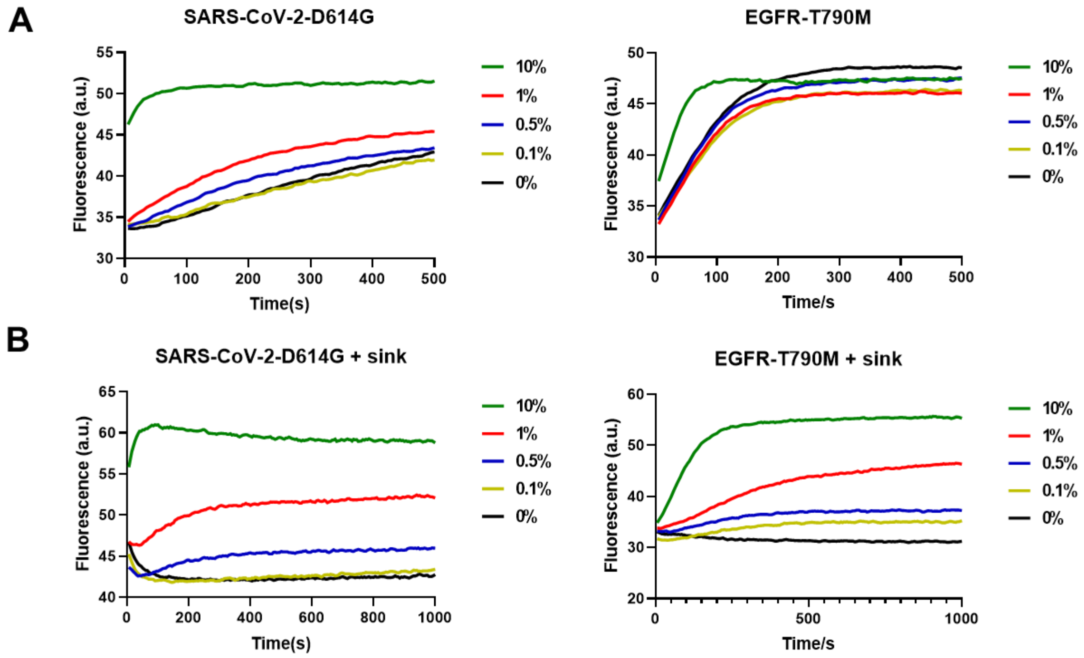

Figure S12. Low VAF detection. (A) Fluorescence curves of TMSD probes (Toe7 mismatch) in the presence of different percentages of SNV strands. (B) Fluorescence curves of probe-sink systems (Toe7 mismatch) in the presence of different percentages of SNV strands.

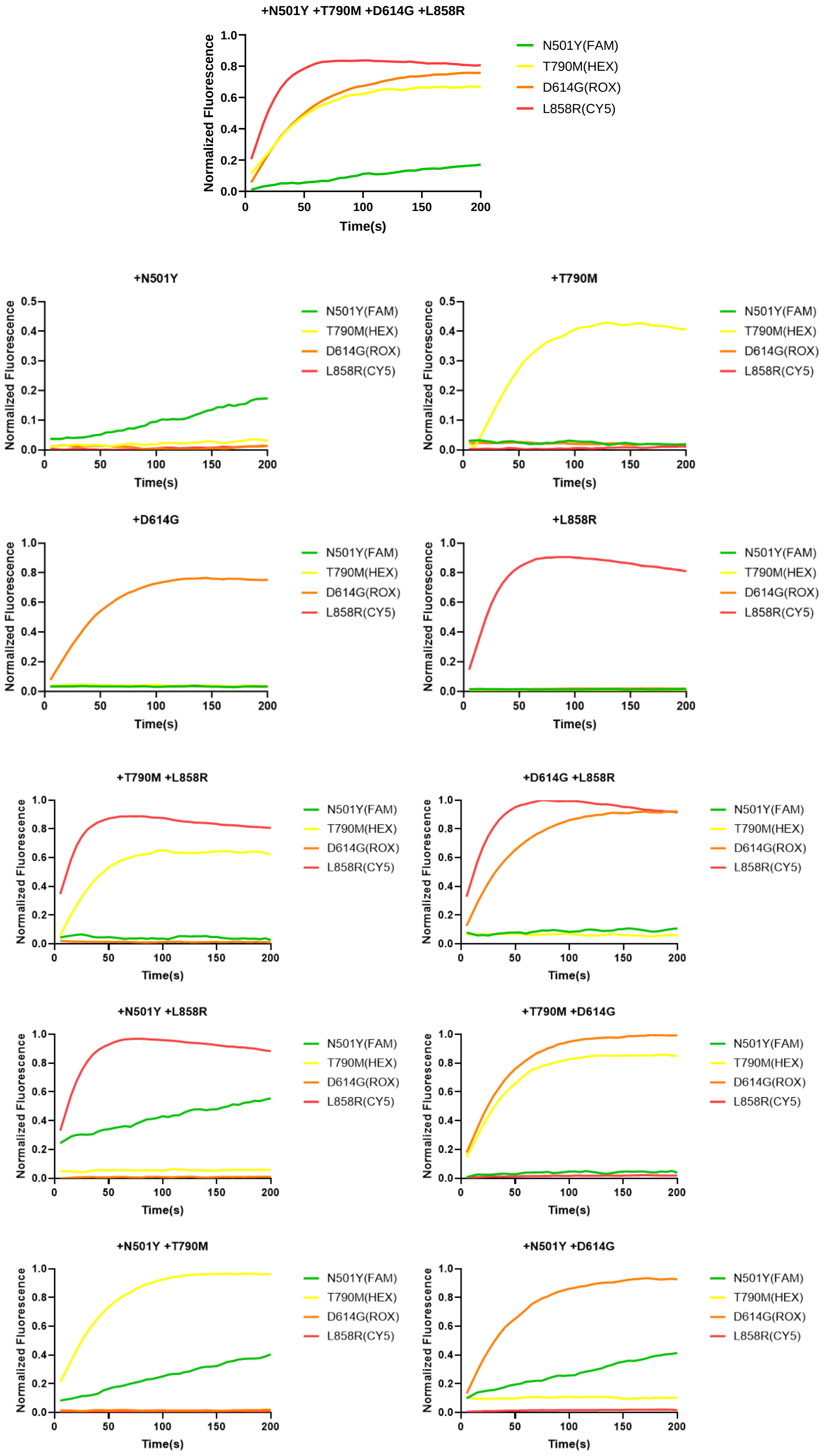
+N501Y +T790M +D614G +L858R
+N501Y
+T790M
+D614G
+L858R
+T790M +L858R
+D614G +L858R
+N501Y +L858R
+T790M +D614G
+N501Y +T790M
+N501Y +D614G
Normalized Fluorescence
Time(s)
N501Y(FAM)
T790M(HEX)
D614G(ROX)
L858R(CY5)

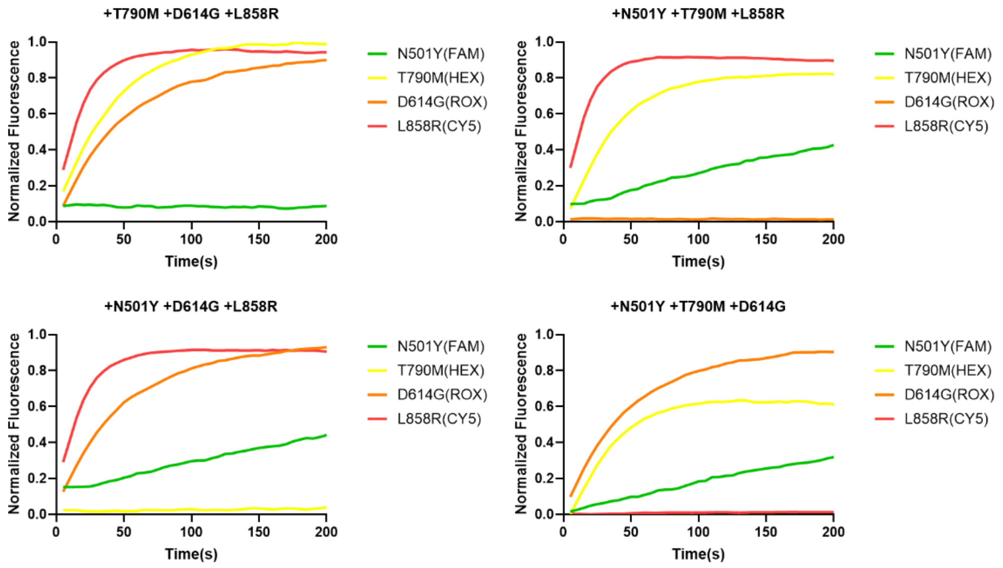

Figure S13. Raw fluorescence curves of multiplex detection. Due to the different excitation efficiencies of the dyes, each color was normalized respectively. The fluorescence intensities of the products and reactants of TMSD reaction were normalized as ‘1’ and ‘0’ for each color.

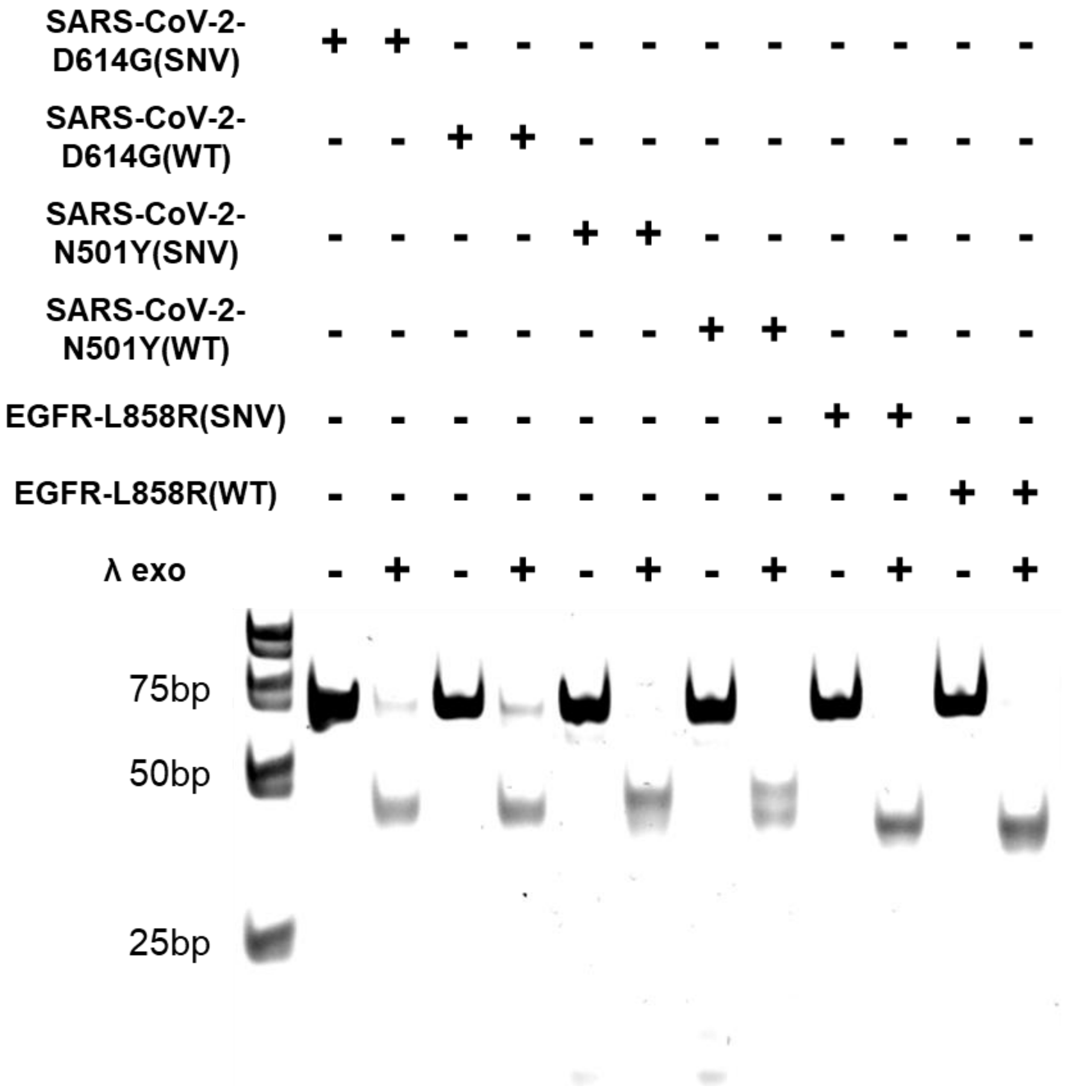

Figure S14. Gel electrophoresis of PCR products from plasmids, and single-stranded PCR generation by λ exonuclease.
